# Global gene expression profiling under nitrogen stress identifies key genes involved in nitrogen stress adaptation in maize (*Zea mays *L.)

**DOI:** 10.1038/s41598-022-07709-z

**Published:** 2022-03-10

**Authors:** Prabha Singh, Krishan Kumar, Abhishek Kumar Jha, Pranjal Yadava, Madan Pal, Sujay Rakshit, Ishwar Singh

**Affiliations:** 1 Indian Council of Agricultural Research-Indian Institute of Maize Research, Pusa Campus, New Delhi, 110012 India; 2 Indian Council of Agricultural Research-Indian Agricultural Research Institute, Pusa Campus, New Delhi, 110012 India; 3 Present Address: Indian Council of Agricultural Research-Indian Grassland and Fodder Research Institute, Jhansi, 284003 India

**Keywords:** Plant biotechnology, Biotechnology, Molecular biology, Plant sciences

## Abstract

Maize is a heavy consumer of fertilizer nitrogen (N) which not only results in the high cost of cultivation but may also lead to environmental pollution. Therefore, there is a need to develop N-use efficient genotypes, a prerequisite for which is a greater understanding of N-deficiency stress adaptation. In this study, comparative transcriptome analysis was performed using leaf and root tissues from contrasting inbred lines, viz., DMI 56 (tolerant to N stress) and DMI 81 (susceptible to N stress) to delineate the differentially expressed genes (DEGs) under low-N stress. The contrasting lines were grown hydroponically in modified Hoagland solution having either sufficient- or deficient-N, followed by high-throughput RNA-sequencing. A total of 8 sequencing libraries were prepared and 88–97% of the sequenced raw reads were mapped to the reference B73 maize genome. Genes with a *p* value ≤ 0.05 and fold change of ≥ 2.0 or ≤ − 2 were considered as DEGs in various combinations performed between susceptible and tolerant genotypes. DEGs were further classified into different functional categories and pathways according to their putative functions. Gene Ontology based annotation of these DEGs identified three different functional categories: biological processes, molecular function, and cellular component. The KEGG and Mapman based analysis revealed that most of the DEGs fall into various metabolic pathways, biosynthesis of secondary metabolites, signal transduction, amino acid metabolism, N-assimilation and metabolism, and starch metabolism. Some of the key genes involved in N uptake (high-affinity nitrate transporter 2.2 and 2.5), N assimilation and metabolism (glutamine synthetase, asparagine synthetase), redox homeostasis (SOD, POX), and transcription factors (MYB36, AP2-EREBP) were found to be highly expressed in the tolerant genotype compared to susceptible one. The candidate genes identified in the present study might be playing a pivotal role in low-N stress adaptation in maize and hence could be useful in augmenting further research on N metabolism and development of N-deficiency tolerant maize cultivars.

## Introduction

Nitrogen (N) is the most important macronutrient required for the growth and development of crop plants. It is a crucial structural element in major biomolecules, viz., chlorophyll, proteins, enzymes, nucleic acids, and various hormones. Besides nutrients, it also acts as a signal molecule. In the soil, it is available in the more common water-soluble nitrate (NO_3_^−^) form, relatively less common ammonium (NH_4_^+^) form and to a lesser extent as proteins, peptides, or amino acids^1^. Being an essential nutrient, N-deficiency causes chlorosis in leaves, especially in lower leaves, restricts bud growth, and reduces overall plant growth^2–4^. Its deficiency at the early vegetative stage of plant life adversely affects crop yield which cannot be reversed by applying N at later stages^5^. Since its availability strongly affects crop productivity, a vast amount of N fertilizers is applied to increase crop yield. However, nitrogen use efficiency (NUE) in cereal crops ranges from 40 to 50%^6,7^. Thus, a significant amount of fertilizer N is leached into the groundwater or evaporated into the environment and thereby, contributes to contamination of ground and surface water, emission of greenhouse gase i.e. nitrous oxide, and also deteriorates soil health^8–12^. Hence, developing crop genotypes with improved NUE would be of prime importance to achieve sustainable agriculture and high productivity under low input conditions with low environmental footprint.

NUE involves efficiency in N uptake, assimilation, remobilization, and utilization. N uptake and metabolism pathways in plants have been well elucidated. The nitrate transporters present in plant root cells—high (NRT1) and low (NRT2) affinity transporters—help in N uptake from the soil, which is then further metabolized to nitrite and ammonium by nitrate reductase (NR) and nitrite reductase (NiR), respectively. This converted ammonium is then incorporated into the organic form (amino acids) by glutamine synthetase (GS) and glutamate synthase/glutamine-2-oxoglutarate aminotransferase (GOGAT) enzymes, also known as GS/GOGAT cycle^13^. In higher plants, there are two types of glutamine synthetase: GS1 isoenzyme, cytosolic form (have 5 isoforms in *Arabidopsis*), and GS2 isoenzyme, present in the plastid. Similarly, two types of glutamate synthase are also reported-ferredoxin-dependent glutamate synthase (Fd-GOGAT), present in the chloroplast in shoots, and NADH-GOGAT, present in root plastids^14^. The GS2 and Fd-GOGAT assimilate ammonium into glutamine and further into glutamate, respectively in the chloroplast, and are essential for survival under photorespiratory conditions^15^. The GS1 assimilates ammonium into glutamine in root cytosol^16,17^. Assimilated N is transported as asparagine, glutamine, aspartate, and glutamate for storage, assimilation, and utilization^18^. The plants’ ability to effectively remobilize N into maturing fruits or grains is very important to NUE^19^, especially in cereal crops where the grain is also economically important.

NUE is a complex trait and to date considerable efforts have been made to understand the molecular basis of plant responses to N and identifying N-responsive genes, and regulatory factors so that their expression could be manipulated for better NUE^20–23^. For instance, in Arabidopsis, microarray studies revealed that expression levels of various genes vary with different concentrations of nitrate both for long-term, and short-term basis^24,25^. In rice, expression analysis of 10,422 genes by microarray revealed a significant difference in the expression level of 471 genes in root tissue^26^. Similarly, in maize, a few studies have been carried out to identify N stress-responsive genes^27–30^. However, a major limitation in most of these studies was that these have been carried out by using a single genotype. Without comparing the transcriptional differences between N-stress tolerant and susceptible genotypes, it is not prudent to separate N-stress tolerant genes from stress-responsive genes.

Maize (*Zea mays* L.) is an important cereal crop for feed, food and industrial raw material. It is the most produced grain in the world. Predominantly, hybrid maize cultivars are cultivated in intensive cropping systems, with high external N input. It is a heavy N consumer and it is highly susceptible to N stress especially in the vegetative stage when uptake and utilization of N are at their peak. To develop N efficient maize genotypes, it is highly essential to delineate the candidate genes and master regulators playing a critical role in NUE in maize. To the best of our knowledge, there are two reports in which contrasting genotypes were used for identification of NUE genes: (1) Chen et al*.*^31^, has reported gene expression changes in response to low nitrogen stress in leaf tissues of contrasting maize inbred lines using the Affymetrix maize genome array, (2) Zamboni et al*.*^32^, has analyzed transcriptional changes in the root of a high and a low nitrogen use efficient maize inbred line in the response to nitrate treatment for 24 h in 7-day old seedling. Both of these studies have their limitations, viz., in the first one, differentially expressed genes (DEGs) in leaf tissue was studied, but not in the root tissue while in the second one, expression changes in root tissue of 7-day old seedling was studied and with a short duration of nitrate treatment. However, understanding of the NUE trait can be further improved by identifying genes in leaf as well as in root tissues at a time under effective treatment (longer duration) for nitrogen stress. Hence, the present study aimed to identify key genes involved in determining nitrogen use efficiency in maize. For this, gene expression was analyzed in root and leaf tissue from contrasting (N-deficiency tolerant and susceptible) tropical maize inbred lines under N-deficiency conditions vis-a-vis control conditions using high-throughput RNA sequencing (RNA-seq) technology.

## Results

### Phenotypic performance of maize genotypes

**Figure 1:**
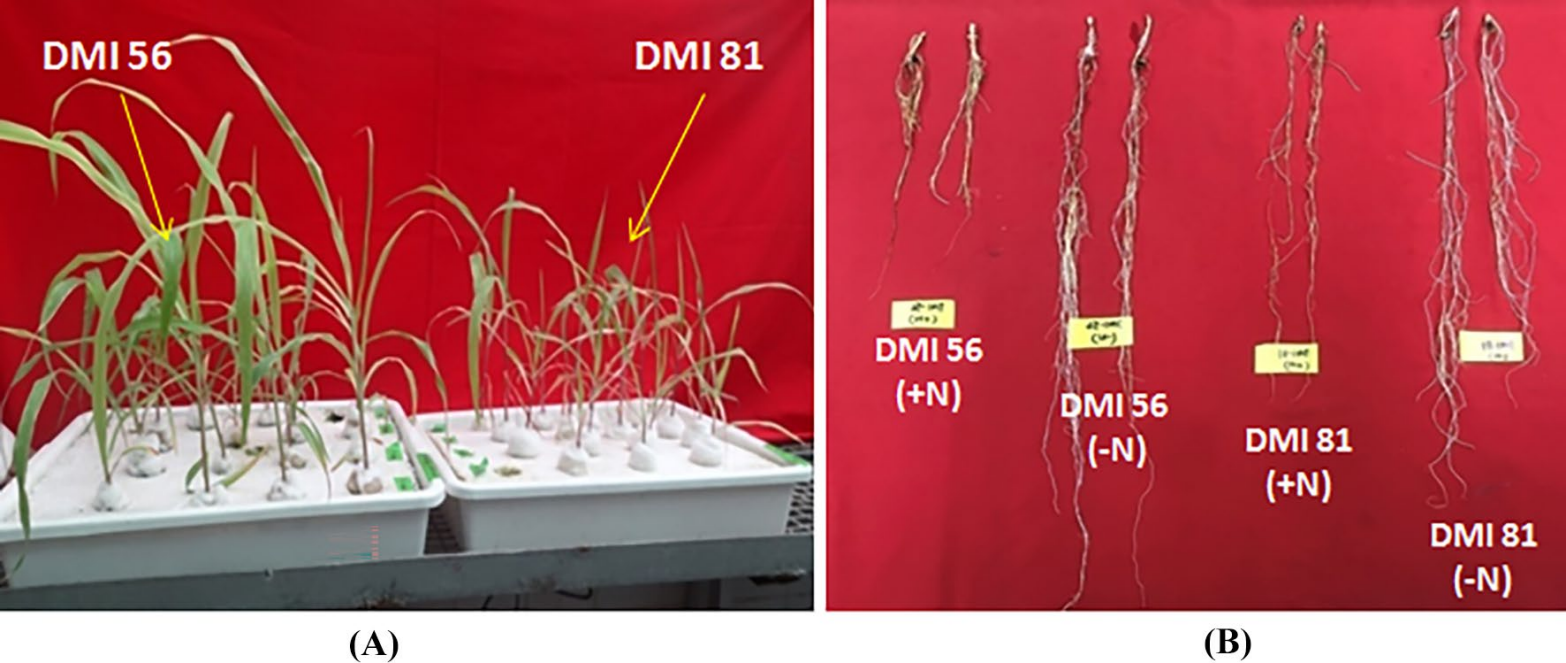
Effect of N-stress on two contrasting maize inbred lines; DMI 56 (tolerance to nitrogen stress) and DMI 81 (susceptible to nitrogen stress) in (**A**) shoot and (**B**) root growth in hydroponic medium. Plants were grown hydroponically under N− deficient (− N) and sufficient/control (+ N) conditions.

Under N-deficiency conditions, the shoot biomass decreased by 56.3% and 68.2% in the N-stress tolerant (DMI 56) and susceptible genotype (DMI 81), respectively, while the root biomass increased significantly in both the genotypes (Table 1, Fig. 1, Supplementary Fig S1). The root of DMI 56 was longer (by 110.8%) when it was grown under low-N for 21 days. The susceptible genotype, though having initially longer roots as compared to the tolerant genotype, could not sufficiently expand its root under N-deficiency (only a 24% increase in root length). Under N-sufficient conditions, the root system of the tolerant genotype was less extensive than those of the susceptible genotype although there was no major difference in the shoot. However, under N-deficiency conditions, the root length of the tolerant genotype was appreciably higher (by 26.8%) than the susceptible genotype. The tolerant genotype had 38.4% greater shoot biomass under N-deficiency as compared to the susceptible genotype. Thus, under N-deficiency conditions, it was found that the tolerant genotype accumulates higher shoot biomass and can dramatically expand its root length.

**Table 1:** Performance (biomass) of seedlings of tolerant (DMI 56) and susceptible genotype (DMI 81) grown under nitrogen sufficient [N (+)] and deficient [N (−)] nutrient solutions for 21 days.

Parameter	DMI 56		DMI 81	
	N (+)	N (−)	N (+)	N (−)
Shoot biomass (g)	7.83 ± 0.087	3.42 ± 0.053	7.77 ± 0.059	2.47 ± 0.071
Root biomass (g)	1.52 ± 0.132	3.23 ± 0.211	1.61 ± 0.093	2.97 ± 0.174
Root length (cm)	34.4 ± 1.5	72.5 ± 3.6	45.7 ± 2.1	56.7 ± 4.8

### Identification of differentially expressed genes under nitrogen-deficiency

**Figure 2:**
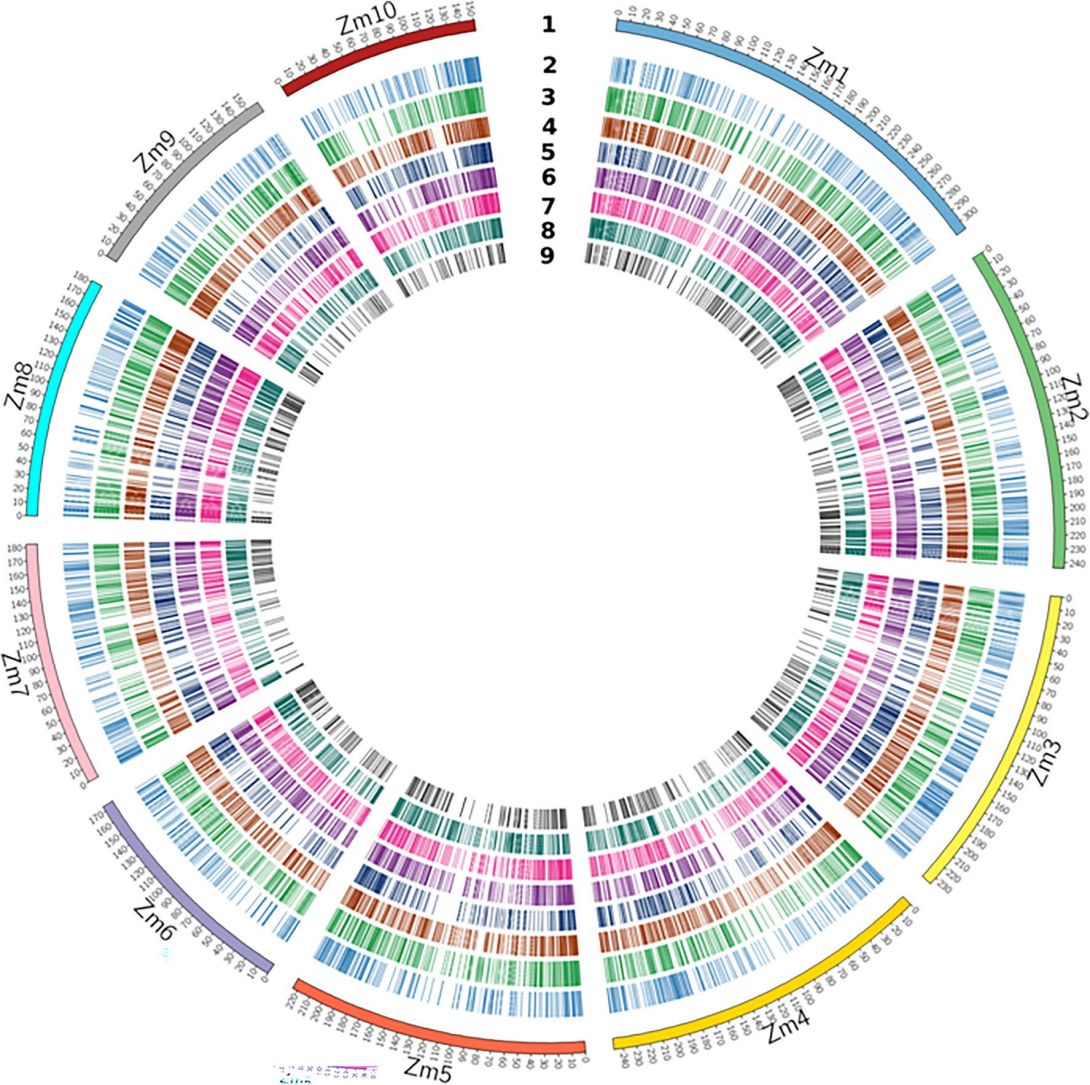
Circos plot depicting the distribution of DEGs on 10 chromosomes of maize. In this figure, all the maize chromosomes are displayed in the first outer ring. 2–9 numbers represents expression (log2 FC) of DEGs in various comparisons, viz., 56_RN+ vs 56_RN−, 56_RN− vs 81_RN−, 56_RN+ vs 81_RN+ , 56_SN+ vs 56_SN−, 56_SN− vs 81_SN−, 56_SN+ vs 81_SN+, 81_RN+ vs 81_RN− and 81_SN+ vs 81_SN− respectively. Letter S, R, N+, and N− represents leaf, root, sufficient nitrogen, and nitrogen-deficiency conditions, respectively. 81 and 56 correspond to DMI 81 (N stress-susceptible genotype) and DMI 56 (N-stress tolerant genotype), respectively.

**Figure 3:**
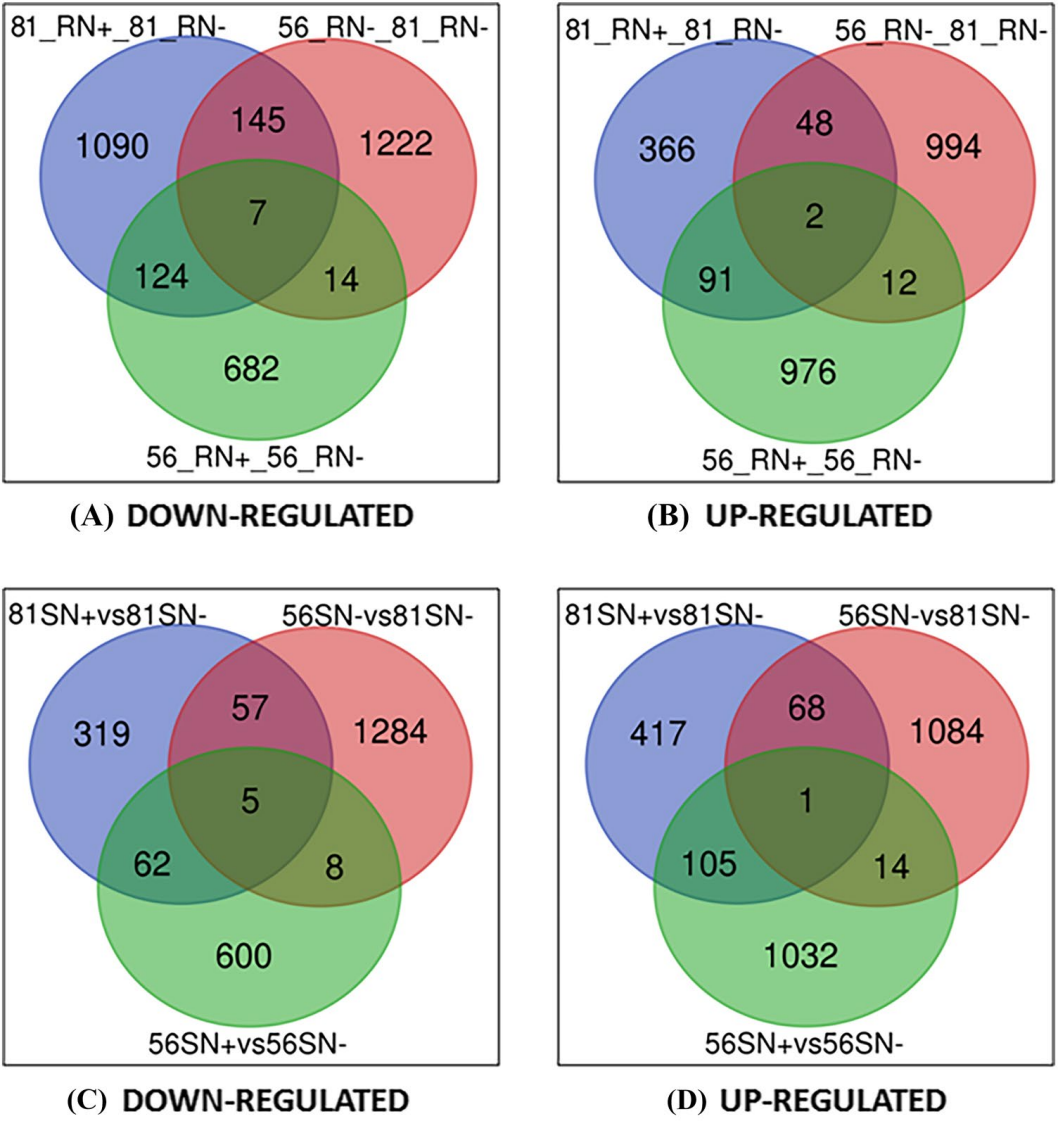
Venn diagram showing the number of down-regulated (**A**,**C**) and up-regulated (**B**,**D**) differentially expressed genes (DEGs) in root and leaf in various combinations. Letter S, R, N+, and N− represents leaf, root, sufficient nitrogen, and nitrogen-deficiency conditions, respectively. 81 and 56 correspond to DMI 81 (N stress-susceptible genotype) and DMI 56 (N-stress tolerant genotype), respectively.

To identify significant DEGs under N-deficiency stress in tropical maize, transcript profiling from contrasting inbred lines was studied under N-deficient and control conditions (see ***“Methods” section for details). As the correct understanding of the differential expression of genes is key to infer phenotypic variations observed among genotypes, therefore, 6 comparisons were made between DMI 56 (tolerant line) and DMI 81(susceptible line) root and leaf tissues to analyze DEGs. A total of 1908, 2444, 1827, 2521, 1873, and 1034 significant DEGs were mapped and annotated in the case of 1st, 2nd 3rd, 4th, 5th, and 6th combinations, respectively (Table 2). A complete list of significant DEGs is provided in Supplementary Table S1 (excel file). The chromosome-wise distribution of DEGs from various combinations was visualized using Circos representation for all the ten chromosomes (Fig. 2). A total of seven and two DEGs in root were common in all three down-regulated and up-regulated combinations, respectively (Fig. 3). Similarly, in leaf, five DEGs were common in all three down-regulated combinations whereas one DEG was found common in up-regulated combinations.

**Table 2:** Comparisons across samples for identification of total and significantly differentially expressed genes under nitrogen stress.

S. No	Comparison	Total genes	Genes filtered at two-fold change	Genes filtered at two-fold change and *p*-value 0.05
1	56_RN+ versus 56_RN−	41,868	6344	1908
2	56_RN− versus 81_RN−	41,853	9478	2444
3	56_SN+ versus 56_SN−	40,514	6276	1827
4	56_SN− versus 81_SN-	41,524	10,200	2521
5	81_RN+ versus 81_RN-	41,309	5866	1873
6	81_SN+ versus 81_SN−	40,724	4713	1034

Letter S, R, N+ , and N− represent leaf, root, sufficient nitrogen, and nitrogen deficiency conditions, respectively. Number 56 and 81 corresponds to DMI 56 and DMI 81 maize inbred lines, respectively.

**Figure 4:**
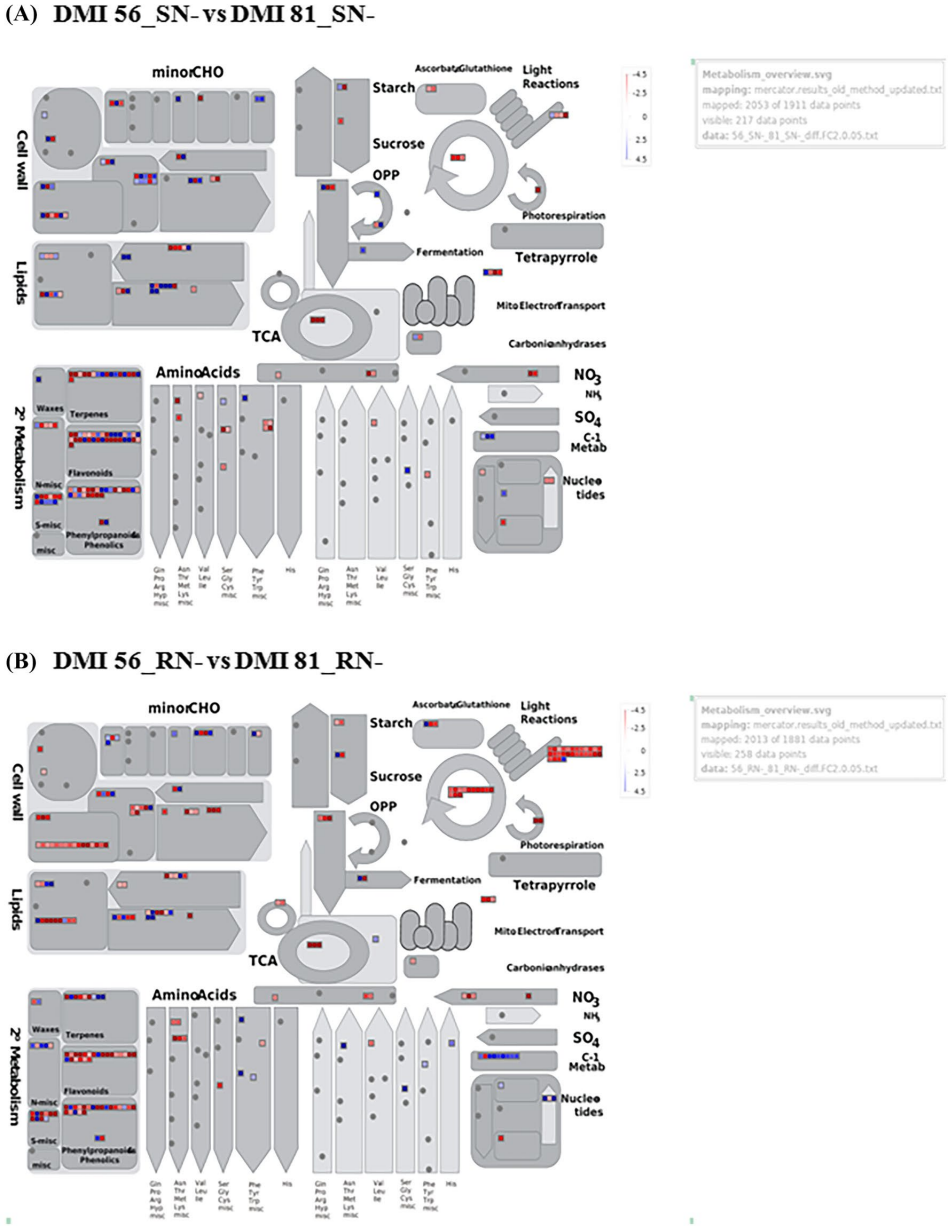
MapMan-based visualization of the DEGs in DMI 81 as compared to DMI 56 under low-nitrogen stress (**A**) leaf (**B**) root. Small blue and red colour squares represent up-and down-regulated genes, respectively at the amplitude of 4.5 to − 4.5 (log2-value).

**Figure 5:**
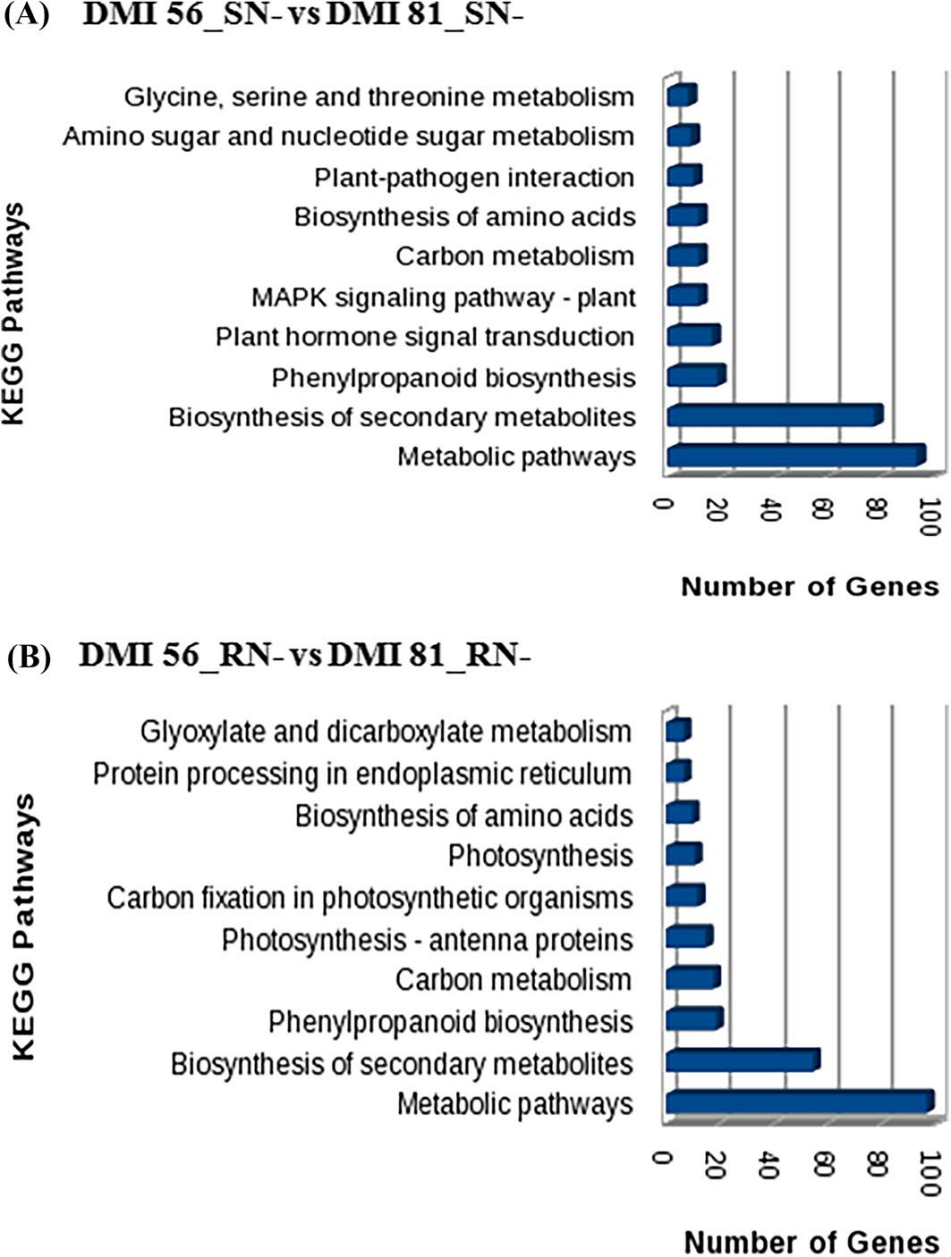
KEGG pathway enrichment analysis of DEGs in comparison (**A**) (DMI 56_SN− vs DMI 81_SN−) and (**B**) (DMI 56_RN− vs DMI 81_RN−) under low-N stress condition. The top 10 pathways from the KEGG enrichment analysis are shown as a bar chart. The terms of the KEGG pathways are depicted on the y-axis. The number of DEGs is shown as the length of the histogram. Letters S, R, and N− represent leaf, root, and nitrogen-deficiency conditions, respectively.

The MapMan based visualization of the expression of DEGs onto metabolic pathways revealed that the maximum number of up-regulated genes were related to secondary metabolism followed by cell wall and lipids processes in DMI 81 leaf as compared to DMI 56 leaf under low-N stress (Fig. 4A). Other than C-1 metabolism, most of DEGs related to photosynthesis, starch-sucrose metabolism, N metabolism were down-regulated. While in the case of root under low-N stress, the maximum number of DEGsbelonged to the cell wall, lipids, and secondary metabolism pathways and most of them showed down-regulation. Besides, DEGs related to NO_3_ metabolism, amino-acid metabolism, and photosynthesis were down-regulated and C-1 metabolism was up-regulated (Fig. 4B). Further, KEGG Pathway analysis revealed that the maximum number of the DEGs could be classified into metabolic pathways, biosynthesis of secondary metabolites, phenylpropanoid biosynthesis in both the comparisons i.e. DMI 81 leaf and root as compared to DMI 56 leaf and root, respectively, under low-N stress (Fig. 5).

**Figure 6:**
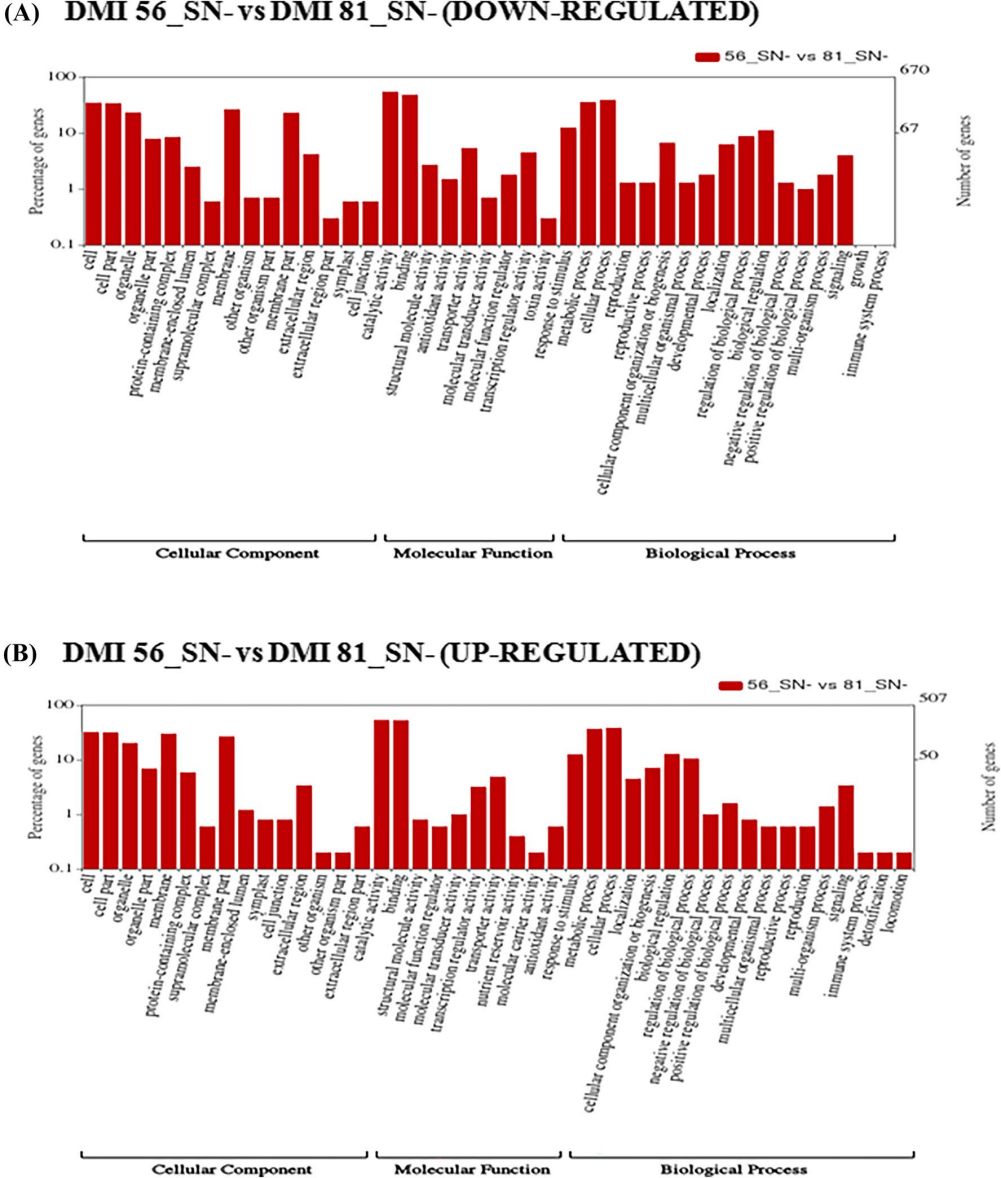
Wego plot for (**A**) down-regulated and (**B**) up-regulated GO classification in accordance to GO groups: molecular function, biological process, and cellular component in DMI 81 leaf compared to DMI 56 leaf under low-N stress (named as DMI 56_SN− vs DMI 81_SN−).

**Figure 7:**
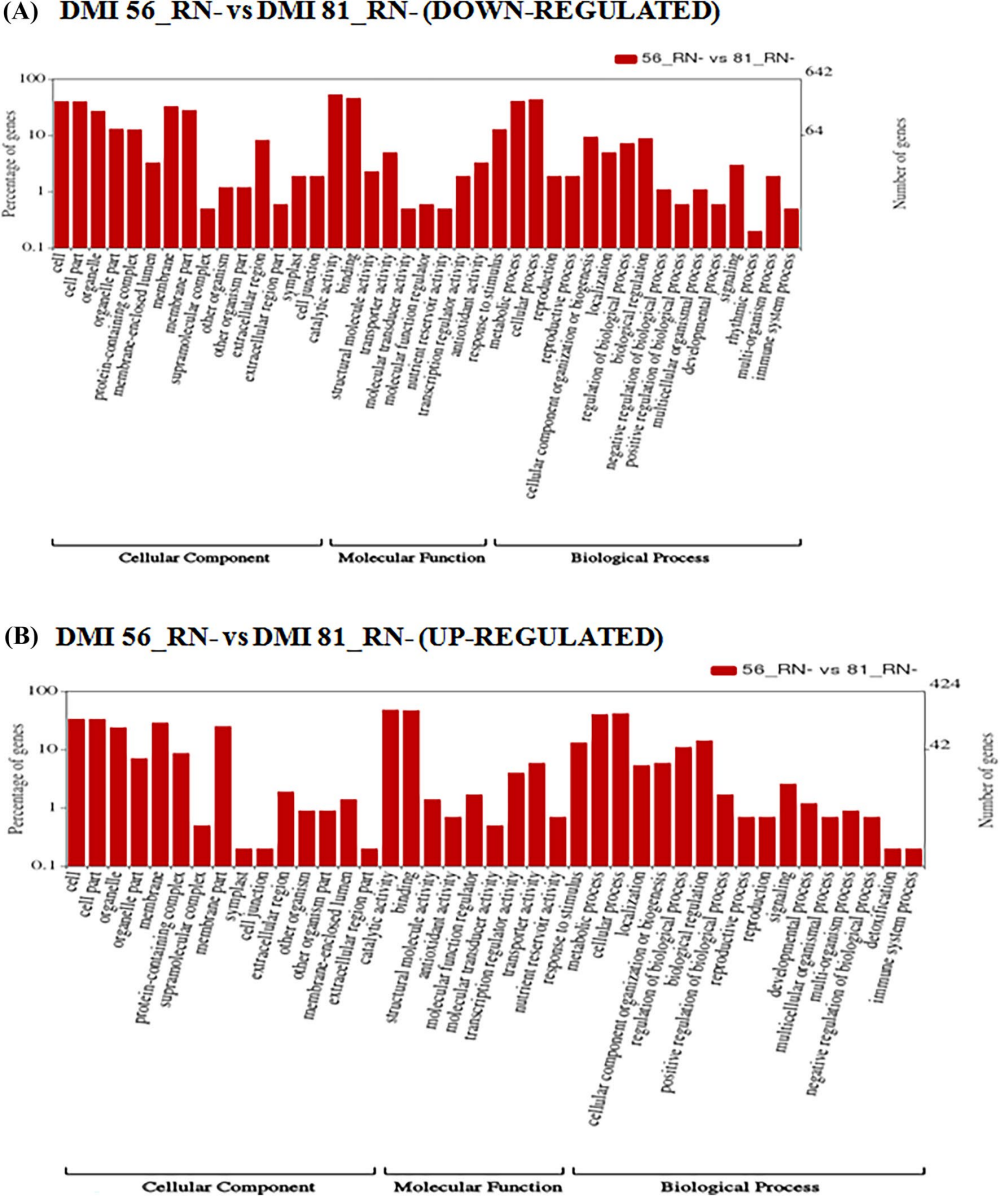
Wego plot for (**A**) down-regulated and (**B**) up-regulated GO classification in accordance to GO groups: molecular function, biological process and cellular component in DMI 81 root as compared to DMI 56 root under low-N stress (DMI 56_RN−_VS_DMI 81_RN−).

WEGO plots represent up-regulated and down-regulated GO annotations which were distributed into three categories namely molecular functions, cellular components, and biological processes. Under low-N stress, a total of 1354 genes in the leaf tissue were down-regulated in the DMI 81 (susceptible) genotype as compared to DMI 56 (tolerant), whereas 1167 genes were up-regulated (Supplementary Table S1 given as excel file). Among the down-regulated genes, most of the genes belonged to biological processes and cellular components followed by molecular function (Fig. 6A). A similar pattern was observed in up-regulated genes (Fig. 6B). When we analyzed the DEGs in the root under low-N stress, 1056 genes were found up-regulated, whereas 1388 were down-regulated in DMI 81 as compared to DMI 56 (Supplementary Table S1). Genes involved in the cellular category and followed by biological process were prominently differentially expressed in the root tissue (Fig. 7).

**Figure 8:**
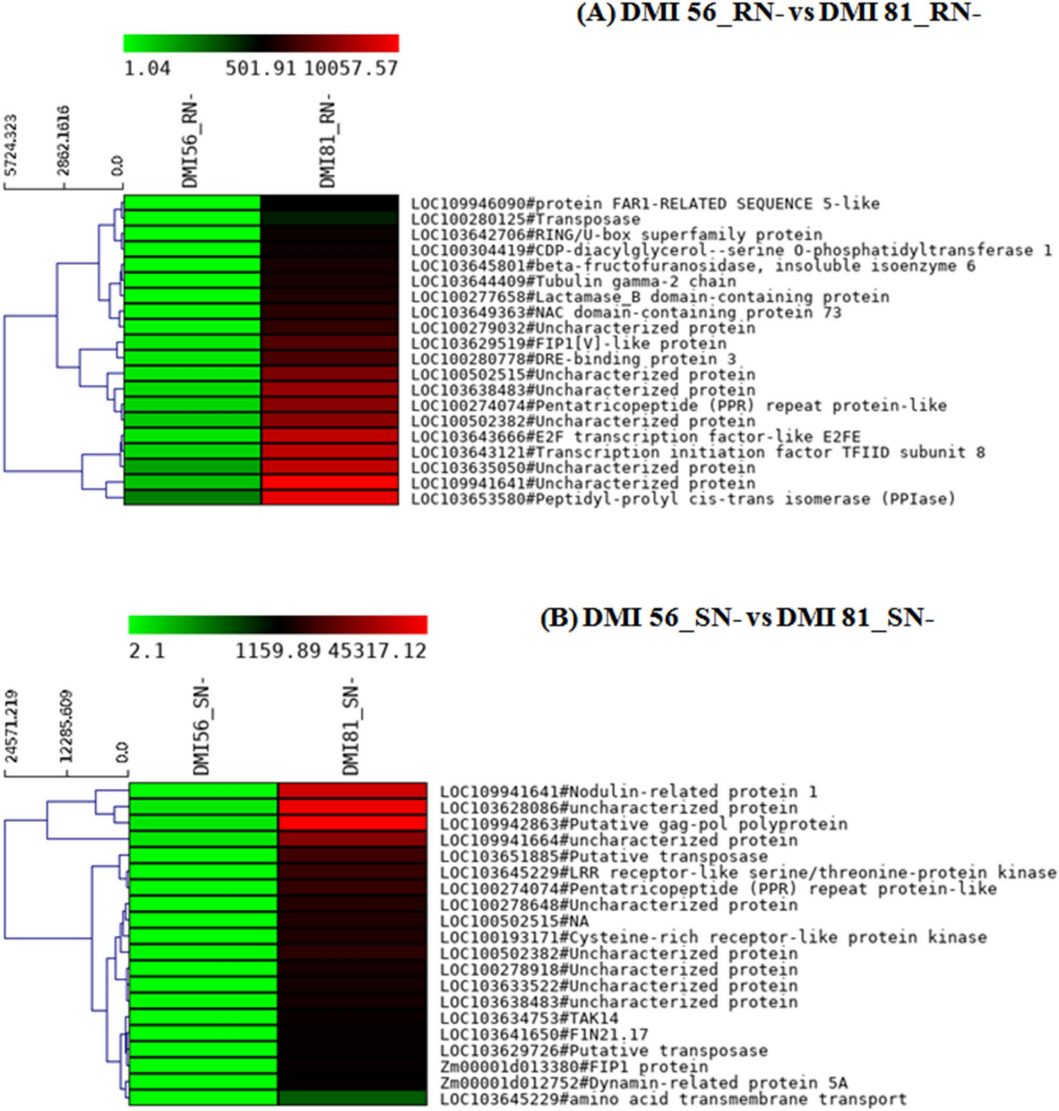
Heat map depicting top 20 down-regulated genes with *p* value < 0.05 in following combinations (**A**) (DMI 56_RN− vs DMI 81_RN−) and (**B**) DMI 56_SN− vs DMI 81_SN−). In the heat maps, each horizontal line refers to a gene. Relatively up-regulated genes are shown in red colour, whereas down-regulated genes are shown in green colour.

**Figure 9:**
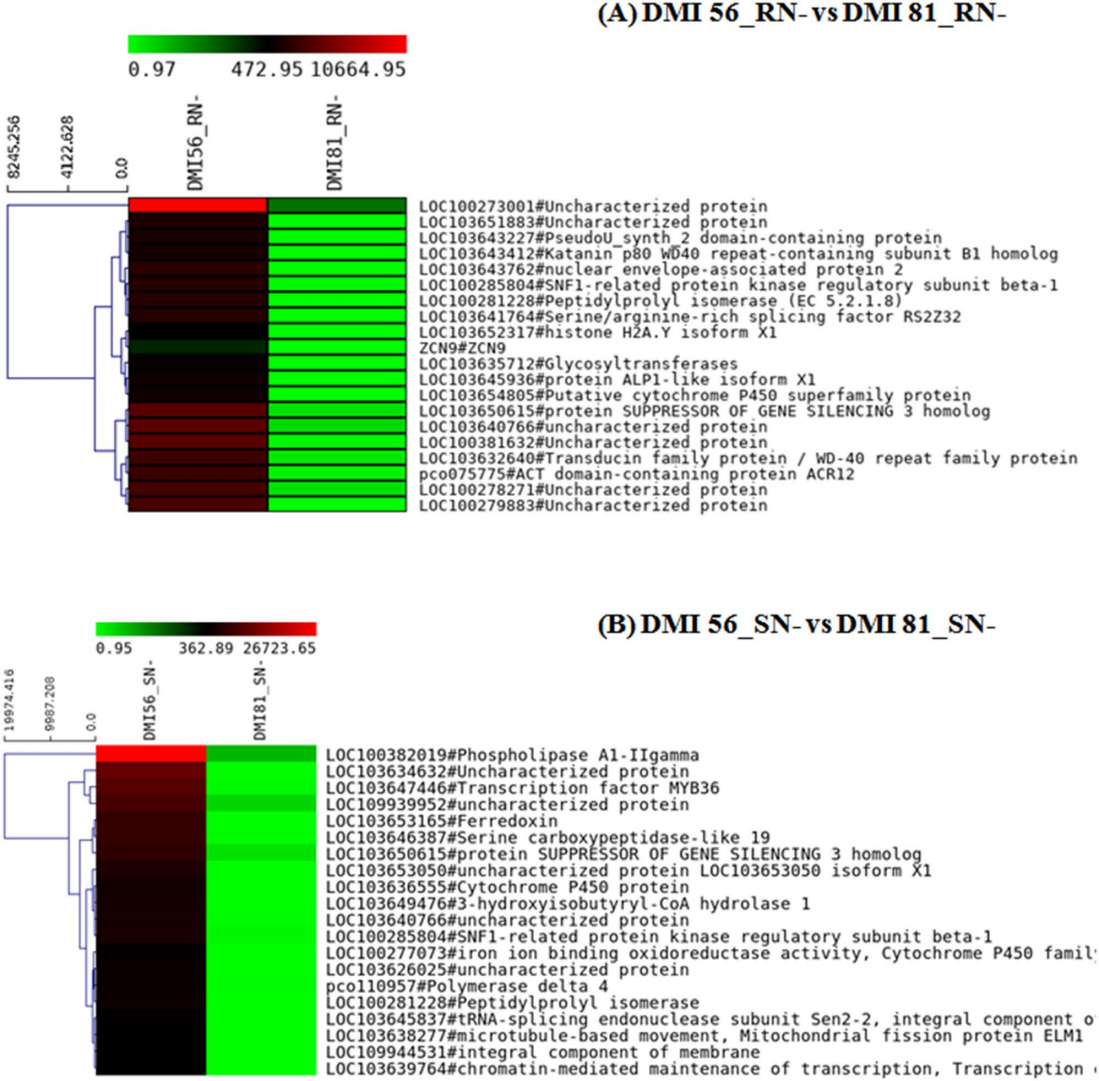
Heat map depicting top 20 up-regulated genes with *p* value < 0.05 in following combinations (**A**) (DMI 56_RN− vs DMI 81_RN−) and (**B**) DMI 56_SN− vs DMI 81_SN−). In the heat maps, each horizontal line refers to a gene. Relatively up-regulated genes are shown in red colour, whereas down-regulated genes are shown in green colour.

Further, top 20 up-regulated and down-regulated genes under N-deficiency in root and leaf tissues of susceptible genotype in comparison to tolerant genotype were shortlisted (Figs. 8, 9). In all combinations, few of the DEGs were uncharacterized which means that their sequence does not match any annotated genes in the database, hence they can be called novel transcripts. Furthermore, the significant DEGs in leaf and root of DMI 56 and DMI 81 under low-N stress conditions compared to their respective control (sufficient N) are summarized in the Supplementary material (Supplementary Figs. S2–S9) while the heat map of top 20 DEGs corresponding to these combinations are given in Supplementary Figs. S10–S13.

### Validation of the expression pattern of the DEG by qRT-PCR

**Figure 10:**
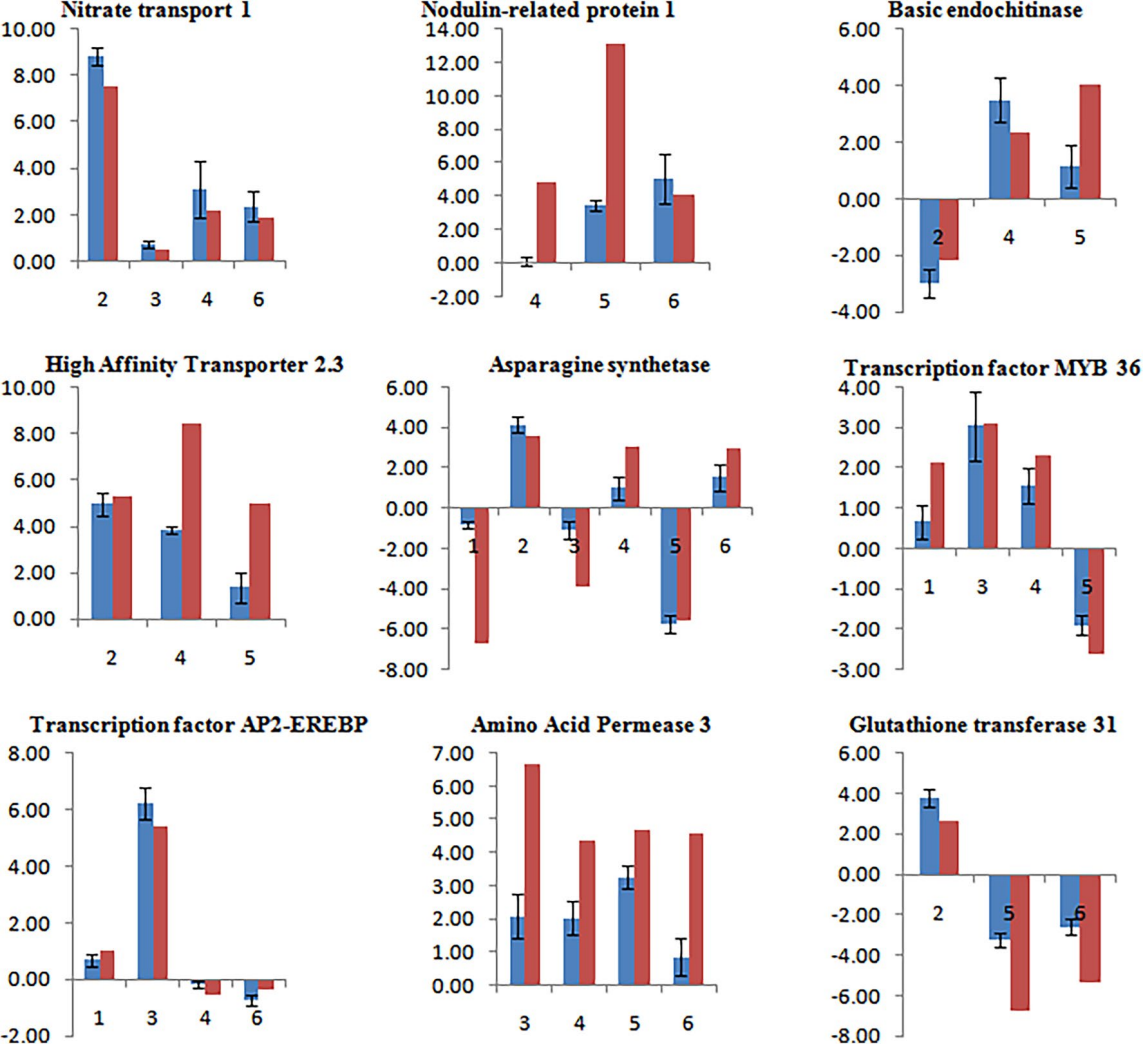
Comparison of expression analysis of selected nitrogen stress-responsive genes via qRT-PCR (represented by blue colour) and NGS approach (represented by red colour) in maize inbreds, viz., DMI 56 (tolerant to nitrogen stress) and DMI 81 (susceptible to nitrogen stress) in response to nitrogen stress treatment. 1–6 numbers on *X*-axis represent comparisons in which a particular gene has significant expression. 1–6 corresponds to DMI 81_SN+ v/s DMI 81_SN−, DMI 81_RN+ v/s DMI 81_RN−, DMI 56_SN+ v/s DMI 56_SN−, DMI 56_RN+ v/s DMI 56_RN−, DMI 56_SN− v/s DMI 81_SN− and DMI 56_RN− v/s DMI 81_RN−, respectively. Letter S, R, N+, and N− represents leaf, root, sufficient nitrogen, and nitrogen-deficiency conditions respectively. *Y*-axis represents the log2 fold change in expression level.

Nine different DEGs were selected based on their function in different pathways to validate the expression pattern via a quantitative real-time polymerase chain reaction (qRT-PCR). The qRT-PCR based expression profiling showed similar gene expression patterns as in Illumina sequencing analysis for all the selected DEGs. Mostly, fold changes obtained by sequencing were higher than those obtained by qRT-PCR. Selected DEGs were: Asn4 (Asparagine synthetase), HAT 2.3 (High-affinity transporter 2.3), NRP1 (Nodulin-related protein 1), basic endochitinase, AAP3 (Amino acid permease 3), GT31 (Glutathione transferase31), MYB 36 transcription factor, AP2-EREBP transcription factor, Nitrate transport 1 (Fig. 10). These key DEGs selected for validation encode genes and transcription factors playing a pivotal role in nitrogen metabolism, transport, and signaling mechanisms. For example, the asparagine synthetase enzyme helps in ammonium assimilation and also plays an important role in nitrogen assimilation, recycling, transport, and storage in plants. Amongst the other DEGs, HAT 2.3 encodes for a high-affinity transporter for nitrogen and nitrate transporter 2.5 is involved in the constitutive high-affinity transport system under long-term N starvation conditions in plants.

## Discussion

The present study was undertaken with the aim to identify key genes playing crucial roles in N stress tolerance in tropical maize. For this, we studied the transcriptome of leaf and root tissues from contrasting maize genotypes under N-deficiency as well as control conditions. Maize plants grown hydroponically under low-N stress conditions exhibited visual symptoms of N-deficiency such as stunted growth, pinkish-red coloration in shoots, yellow coloration in older leaves, upright leaves with light green/yellow color, and burnt leaf margin, etc. These symptoms were more prominent in the susceptible genotype as compared to the tolerant one (Supplementary Fig S1). Soltabayeva et al*.*^33^ have reported accelerated yellowing and senescence of old leaves as one of the typical symptoms of N-deficiency in plants. Further, under low-N stress conditions, a significant reduction in shoot biomass was observed in both susceptible and tolerant genotypes (Table 1). This indicates stress-related growth retardation, highlighting the prominent role of N for biomass accumulation. However, there was a pronounced increase in root length under low-N stress conditions as compared to N-sufficient conditions (Fig. 1; Table 1). Importantly, the percentage increase in root length in the tolerant genotype was much higher than the susceptible genotype. The increase in root length indicates the adaptive response of maize plants under N-limitation to maximize N uptake by increasing the surface area for acquisition. Further, it suggests that the tolerant genotype has a better potential to adapt under low-N stress conditions as compared to the susceptible genotype. A similar pattern of results has been reported in N-deficiency conditions after 15 days of treatment in wheat^34^.

### Transcriptome analysis and genes responsible for nitrogen-deficiency stress adaptation

Transcriptomics is an important and powerful approach being used for global gene expression profiling in different crop plants under abiotic, biotic, and nutrient deficiency stresses^21–23,35,36^. In the present study, transcriptomic comparison of low-N stress-tolerant (DMI 56) and susceptible (DMI 81) genotypes was attempted to delineate potential candidate genes involved in N-deficiency stress in tropical maize. The cDNA samples from root and leaf tissues were sequenced and 40,724–41,868 total genes were identified, out of which significant genes ranged from 1034 to 2521 in six different comparisons (Table 2). The number of total genes and significant genes identified in the present study were comparable to other studies^31,37,38^. The number of DEGs in both root and leaf tissues were higher in the tolerant genotype (DMI 56) as compared to the susceptible genotype (DMI 81) under N stress (Table 2). Further, it was observed that in the tolerant genotype, the number of up-regulated genes was more than the down-regulated genes as compared to the susceptible genotype (Fig. 3). In various comparisons, the number of down-regulated genes ranged from 827 to 1388 in root and 443 to 1354 in leaf while the number of up-regulated genes ranged from 507 to 1081 in root and 591 to 1167 in leaf (Fig. 3; Supplementary Table S1). The up-regulated and down-regulated genes obtained in our study are comparable to those reported recently in Tibetan hulless barley^39^ and sorghum^40^ but lower than rice^41^ under N stress.

Our study revealed that most of the DEGs were mainly confined to secondary metabolism, cell-wall, and lipid component in all six combinations (Fig. 4; Supplementary Figs. S2, S3). Some DEGs also showed significant up-regulation and down-regulation in C-1 metabolism, NO_3_ metabolism, starch sucrose cycle, photosynthesis, amino-acid metabolism, and photorespiration. Interestingly, most of the DEGs were up-regulated and down-regulated in root tissues of DMI 56 and DMI 81, respectively, under low-N stress as compared to their respective control (Supplementary Figs. S2, S3). Further, the number of DEGs was more in root than in the leaf. In *Arabidopsis thaliana,* DEGs associated with the above-mentioned processes played a very important role in adaptation under low-N stress^42^. Similarly, our finding also corroborates with previously identified DEGs related to various pathways in rice^43^ and wheat under varied nitrogen supplies^44^.

Different studies have reported the involvement of genes related to amino acid metabolism, lipid metabolism, energy metabolism, and signal transduction pathways for tolerance against N stress^45–47^. For example, in sorghum roots, under low-N stress, DEGs associated with amino-acid metabolism play a key role^40^. Similarly, protein kinases (PK) are prominently involved in response to N stress in *Arabidopsis thaliana* leaves and roots^44^. In this study, genes related to metabolic pathways, biosynthesis of secondary metabolite, plant hormone signal, biosynthesis of amino acids, photosynthesis, MAPK signaling, etc. were identified in both root and leaves (Fig. 5; Supplementary Figs. S4, S5). The present findings support the results from previous studies and confirm that all these pathways form a complex network in N-stress adaptation in plants.

### Key differentially expressed genes potentially involved in determining NUE in maize

In the current study, genes that showed the highest up-regulation in susceptible line root tissue compared to the tolerant line root tissue under low-N stress conditions are beta-fructofuranosidase (LOC103645801), protein FAR1-RELATED SEQUENCE 5-like (LOC103651448), tubulin gamma-2 chain (Zm00001d027568), RING/U-box superfamily protein (Zm00001d025230), CDP-diacylglycerol–serine O-phosphatidyltransferase 1 (Zm00001d018013), transposase, etc. (Table 3). Beta-fructofuranosidase or invertase can hydrolyze sucrose into glucose and fructose and have been shown to play role in regulating the growth and development of plants under biotic and abiotic stresses^48^. Protein FAR1-RELATED SEQUENCE 5-like encode transposase-derived transcription factors, which played important roles in phosphate stress^28^, oxidative response^49^, chlorophyll biosynthesis^50^, starch synthesis^51^. Tubulin gamma-2 chain is essential for acentrosomal microtubule nucleation which is crucial for cell division^52^. RING/U-box superfamily protein is a class of E3 ligases family and it has been reported that it plays important role in N stress response in Arabidopsis^25^. Transposons have shown stress-responsive expression and/or transposition in many crops such as in tobacco under biotic and abiotic stress^53^, in rice under cold and salt stress^54^ and Arabidopsis under heat stress^55^. CDP-diacylglycerol–serine O-phosphatidyltransferase catalyzes base-exchange reaction where phosphatidylcholine is replaced by serine and is involved in amino acid metabolism^56^.

**Table 3:** Selected top 20 up- and down-regulated genes in DMI 81 root as compared to DMI 56 root under low-nitrogen stress (DMI 56_RN− vs DMI 81_RN−) using EdgeR.

DMI 56_RN− vs DMI 81_RN−					
ID	Gene I’D	Log_2_ fold change	*p* value	FDR	Gene description
NC_024462.2:208046149–208049422(−)	LOC109946090	9.08	3.10E−06	0.000595	Protein FAR1-RELATED SEQUENCE 5-like
NC_024459.2:252552782–252556786(+)	LOC103643666	7.22	1.11E−15	9.31E−12	E2F transcription factor-like E2FE
NC_024468.2:110222626–110223440(+)	LOC103642706	8.86	2.04E−08	8.87E−06	RING/U-box superfamily protein
NC_024468.2:41411289–41412420(+)	LOC100280125	8.74	1.97E−05	0.002659	Transposase
NC_024466.2:34002872–34004339(+)	LOC109941641	6.37	5.96E−14	2.76E−10	Uncharacterized protein
NC_024459.2:200066866–200075746(−)	LOC103643121	6.52	6.58E−14	2.76E−10	Transcription initiation factor TFIID subunit 8
NC_024460.2:82663571–82667472(−)	LOC100502515	6.69	2.32E−13	7.93E−10	Uncharacterized protein
NC_024467.2:68904906–68908763(−)	LOC103638483	6.47	2.46E−13	7.93E−10	Uncharacterized protein
NC_024466.2:178362960–178366314(+)	LOC100274074	6.11	2.88E−12	7.53E−09	Pentatricopeptide (PPR) repeat protein-like
NC_024461.2:169441733–169448925(+)	LOC100502382	5.79	1.56E−11	3.27E−08	Uncharacterized protein
NC_024460.2:58484696–58490087(−)	LOC100277658	8.66	1.95E−11	3.88E−08	Lactamase_B domain-containing protein
NC_024460.2:226783155–226784854(−)	LOC103649363	7.41	3.04E−11	5.53E−08	NAC domain-containing protein 73
NC_024464.2:80908555–80911518(−)	LOC103629519	6.18	3.43E−11	5.73E−08	FIP1[V]-like protein
NC_024460.2:3244639–3247424(+)	LOC103645801	9.45	3.67E−11	5.91E−08	Beta-fructofuranosidase, insoluble isoenzyme 6
NC_024464.2:40555851–40557027(−)	LOC100279032	6.99	3.92E−11	6.07E−08	Uncharacterized protein
NC_024466.2:34010116–34012763(+)	LOC103635050	5.41	4.49E−11	6.71E−08	Uncharacterized protein
NC_024459.2:8237878–8238802(−)	LOC103644409	8.92	4.97E−11	7.18E−08	Tubulin gamma-2 chain
NC_024465.2:146034581–146035618(−)	LOC100280778	6.16	7.90E−11	1.02E−07	DRE-binding protein 3
NC_024463.2:212102747–212106156(+)	LOC100304419	8.29	4.02E−08	1.54E−05	CDP-diacylglycerol–serine O-phosphatidyltransferase 1
NC_024462.2:107860703–107861642(+)	LOC103653580	5.18	1.12E−10	1.30E−07	Peptidyl-prolyl cis–trans isomerase (PPIase)
**Down-regulated**					
NC_024462.2:235421283–235423392(−)	LOC103654805	− 9.18	1.62E−09	1.02E−06	Putative cytochrome P450 superfamily protein
NC_024460.2:9999124–10002995(+)	LOC103645936	− 9.11	3.62E−09	1.97E−06	Protein ALP1-like isoform X1
NC_024462.2:243980189–243981610(+)	LOC100381632	− 8.32	6.66E−16	6.97E−12	Uncharacterized protein
NC_024466.2:113319211–113321231(−)	LOC103635712	− 8.79	7.03E−08	2.51E−05	Glycosyltransferases
NC_024460.2:230848675–230856504(−)	LOC103643762	− 8.87	1.19E−13	4.52E−10	Nuclear envelope-associated protein 2
NC_024468.2:126118262–126120658(+)	LOC103641764	− 9.03	4.13E−13	1.24E−09	Serine/arginine-rich splicing factor RS2Z32
NC_024466.2:167543593–167547576(+)	LOC100281228	− 8.74	6.77E−13	1.89E−09	Peptidylprolyl isomerase (EC 5.2.1.8)
NC_024465.2:178358779–178366537(−)	LOC100279883	− 11.78	6.65E−19	2.78E−14	Uncharacterized protein
NC_024461.2:235584839–235589281(−)	pco075775	− 6.85	1.30E−11	2.86E−08	ACT domain-containing protein ACR12
NC_024461.2:138093686–138100193(−)	LOC103650615	− 6.12	2.46E−11	4.68E−08	Protein SUPPRESSOR OF GENE SILENCING 3 homolog
NC_024465.2:181066359–181070124(−)	LOC100273001	− 5.33	3.33E−11	5.73E−08	Uncharacterized protein
NC_024466.2:8814413–8820091(+)	ZCN9	− 7.71	6.67E−05	0.007227	ZCN9
NC_024459.2:231626104–231633490(+)	LOC103643412	− 8.96	7.11E−11	9.60E−08	Katanin p80 WD40 repeat-containing subunit B1 homolog
NC_024466.2:123263353–123266407(−)	LOC100285804	− 7.31	8.06E−11	1.02E−07	SNF1-related protein kinase regulatory subunit beta-1
NC_024468.2:5608403–5612399(−)	LOC103640766	− 5.83	8.98E−11	1.07E−07	Uncharacterized protein
NC_024461.2:158634595–158636625(+)	LOC103652317	− 7.54	1.65E−05	0.002318	Histone H2A.Y isoform X1
NC_024461.2:38296249–38300721(−)	LOC103651883	− 7.73	2.36E−10	2.35E−07	Uncharacterized protein
NC_024465.2:101004710–101009817(−)	LOC103632640	− 6.08	2.50E−10	2.40E−07	Transducin family protein / WD-40 repeat family protein
NC_024459.2:209998774–210003204(−)	LOC103643227	− 7.42	1.75E−09	1.07E−06	PseudoU_synth_2 domain-containing protein
NC_024459.2:282017694–282021447(+)	LOC100278271	− 5.82	3.94E−10	3.44E−07	Uncharacterized protein

Letters R, and N- represent root, and nitrogen-deficiency conditions, respectively.

Some of the prominent down-regulated genes in susceptible line root compared to tolerant line root are Putative cytochrome P450 protein (Zm00001d053586), protein ALP1-like isoform X1 (LOC103645936), Serine/arginine-rich splicing factor RS2Z32 (Zm00001d037543), Katanin p80 WD40 repeat-containing subunit B1 homolog (Zm00001d032598), Glycosyltransferases (Zm00001d038981), Peptidylprolyl isomerase (Table 3). The cytochrome P450 (CYP) is involved in various metabolic pathways and performs an important function in metabolic reactions leading to accumulation of secondary metabolites that protect the plant from many biotic and abiotic stresses^41,57^. Thus, down-regulation of this gene under low-N stress may contribute to the susceptible nature of any genotype while up-regulation may lead to tolerant nature. Protein ALP1-like isoform X1 is a type of transposase having nuclease activity and has been reported to provide tolerance under drought stress^58^. SR (serine/arginine-rich) proteins are a highly conserved family of RNA-binding proteins which play a crucial role in pre-mRNA splicing. This RNA splicing has been shown to be tissue-specific, developmentally regulated, and stress-responsive^59^. Katanin is a microtubule-severing protein that regulates cell division and its orientation during plant growth. Its working is under hormonal control^60^. Glycosyltransferase has been demonstrated to help in maintaining membrane integrity under abiotic stress conditions especially during chilling stress^61^. Peptidylprolyl isomerase is a domain in cyclophilins, a ubiquitous protein, which is involved in a wide range of cellular processes such as signaling, cell division, transcriptional regulation under various abiotic stresses^62^. Since these genes have been shown to involve in abiotic or biotic stress tolerance previously in different plants, therefore their up-regulation might play important role in low-N stress tolerance.

Similarly, few genes showing maximum up-regulation in the susceptible line leaf tissue compared to tolerant line leaf tissue under low-N stress are the Nodulin-related protein 1 (Zm00001d008397), LRR receptor-like serine/threonine-protein kinase (Zm00001d051093), Cysteine-rich receptor-like protein kinase (Zm00001d008488), Putative transposase, FIP1 protein (Zm00001d013380) (Table 4). Nodulin-related protein is expressed in Arabidopsis plant parts in all developmental stages and modulated by external environmental cues^63^. Leucine-rich repeats kinases are the largest subgroup of the RLK family with 309 members in rice and 235 members in Arabidopsis and have a very important role in plant response to abiotic stress^64–66^. Similarly, Cysteine-rich receptor-like protein kinases has been shown to be up-regulated under N-deficiency stress in rice^41^. In our study, transposases showed significant up-regulation in root and leaf tissues in susceptible line and down-regulation in the tolerant line under low-N stress conditions. In *Arabidopsis thaliana*, FIP1 (FtsH5 Interacting Protein) protein expression is modulated by light stress and it has been reported that under high light intensity, oxidative, salt, and osmotic stress its expression is downregulated^67^*.* In our study, the DEGs exhibiting maximum down-regulation in leaf tissue under low-nitrogen stress are Ferredoxin (Zm00001d049732), Cytochrome P450 protein, Serine carboxypeptidase-like 19 (Zm00001d003530), Transcription factor MYB36 (Zm00001d005784), Peptidylprolylisomerase (Table 4; Fig. 10). Ferredoxin is related to nitrate/nitrite assimilation, ferredoxin reduction, and the pentose phosphate pathway and has been shown to be downregulated in rice under N-deficiency stress^41^. Further, the top 20 up-and down-regulated DEGs in leaf and root tissues of DMI 56 and DMI 81 under low-N stress conditions compared to respective control are given in Supplementary Table S2–S9.

**Table 4:** Selected top 20 up- and down-regulated genes in DMI 81 leaf as compared to DMI 56 leaf under low-nitrogen stress (DMI 56_SN− vs DMI 81_SN−). Letters S, and N− represent leaf, and nitrogen-deficiency conditions, respectively.

DMI 56_SN− vs DMI 81_SN−						
ID	Gene I’D	Log_2_ fold change		p value	FDR	Gene description
NC_024462.2:141790700–141794379(−)	LOC103645229	12.31		2.77E−22	5.75E−18	LRR receptor-like serine/threonine-protein kinase
NC_024467.2:95501952–95503004(−)	LOC100278648	11.86		3.44E−21	4.76E−17	Uncharacterized protein
NC_024460.2:82663571–82667472(−)	LOC100502515	11.74		8.00E−21	6.64E−17	NA
NC_024466.2:7232773–7233598(+)	LOC109941641	13.11		4.95E−21	5.14E−17	Nodulin-related protein 1
NC_024466.2:9999352–10008136(−)	LOC100193171	10.98		9.92E−20	5.15E−16	Cysteine-rich receptor-like protein kinase
NC_024465.2:176516324–176517425(−)	LOC103633522	11.17		7.57E−19	3.14E−15	Uncharacterized protein
NC_024465.2:176489782–176490883(−)	LOC100278918	10.32		2.09E−18	6.69E−15	Uncharacterized protein
NC_024461.2:38407828–38413383(−)	LOC103651885	9.36		3.36E−18	9.98E−15	Putative transposase
NC_024466.2:178362960–178366314(+)	LOC100274074	8.96		2.01E−17	5.55E−14	Pentatricopeptide (PPR) repeat protein-like
NC_024466.2:9350129–9352712(−)	LOC103634753	9.57		5.50E−15	1.27E−11	TAK14
NC_024461.2:169441733–169448925(+)	LOC100502382	7.77		6.74E−15	1.47E−11	Uncharacterized protein
NC_024464.2:80908555–80911518(−)	Zm00001d013380	9.14		8.25E−13	9.01E−10	FIP1 protein
NC_024468.2:118183614–118183895(−)	LOC103641650	10.30		1.54E−14	3.04E−11	F1N21.17
NC_024464.2:101460979–101461802(−)	LOC103629726	10.24		3.36E−14	5.81E−11	Putative transposase
NC_024466.2:102778468–102780293(−)	LOC109941664	7.75		8.68E−14	1.25E−10	Uncharacterized protein
NC_024467.2:68904906–68908763(−)	LOC103638483	7.61		1.08E−13	1.50E−10	Uncharacterized protein
NC_024463.2:26101030–26104294(+)	LOC103628086	8.69		1.35E−13	1.81E−10	Uncharacterized protein
NC_024468.2:91778559–91792902(+)	LOC109942863	8.63		3.22E−13	3.82E−10	Putative gag-pol polyprotein
NC_024459.2:206905353–206916888(+)	LOC103645229	5.30		0.000175	0.014794	Amino acid transmembrane transport
NC_024463.2:197362553–197368786(−)	Zm00001d012752	8.96		2.47E−10	1.74E−07	Dynamin-related protein 5A
**Down-regulated**						
NC_024459.2:57821705–57827992(−)	LOC103634632		− 12.49	1.05E−22	4.35E−18	Uncharacterized protein
NC_024462.2:41916032–41916802(−)	LOC103653165		− 11.62	2.49E−20	1.72E−16	Ferredoxin
NC_024460.2:188225340–188227618(−)	LOC103647446		− 10.06	2.08E−19	9.60E−16	Transcription factor MYB36
NC_024460.2:46804324–46808360(+)	LOC103646387		− 10.25	1.00E−18	3.58E−15	Serine carboxypeptidase-like 19
NC_024460.2:4925676–4928422(+)	LOC103645837		− 9.23	3.76E−06	0.000643	tRNA-splicing endonuclease subunit Sen2-2, integral component of membrane
NC_024466.2:22354741–22355835(+)	LOC103626025		− 10.61	3.92E−11	3.01E−08	Uncharacterized protein
NC_024462.2:32017164–32018504(−)	LOC103653050		− 11.79	2.72E−17	7.06E−14	Uncharacterized protein LOC103653050 isoform X1
NC_024460.2:27421774–27426376(−)	LOC100277073		− 8.55	2.85E−08	1.03E−05	Iron ion binding oxidoreductase activity, Cytochrome P450 family 72 subfamily A polypeptide 8
NC_024467.2:37432851–37435462(+)	LOC103638277		− 8.00	3.16E−05	0.003664	Microtubule-based movement, Mitochondrial fission protein ELM1
NC_024466.2:175694170–175696689(+)	LOC103636555		− 11.22	3.06E−14	5.52E−11	Cytochrome P450 protein
NC_024466.2:137537911–137539188(+)	LOC100382019		− 8.04	4.08E−14	6.78E−11	Phospholipase A1-IIgamma
NC_024465.2:144351339–144353438(+)	pco110957		− 7.66	1.65E−08	6.67E−06	Polymerase delta 4
NC_024460.2:238320268–238325376(−)	LOC103649476		− 9.76	7.93E−14	1.22E−10	3-Hydroxyisobutyryl-CoA hydrolase 1
NC_024460.2:104888362–104889334(−)	LOC109944531		− 7.57	0.000229	0.018515	Integral component of membrane
NC_024468.2:5608403–5612399(−)	LOC103640766		− 8.89	1.98E−13	2.57E−10	Uncharacterized protein
NC_024466.2:167543593–167547576(+)	–		− 9.84	1.09E−11	9.39E−09	Peptidylprolyl isomerase
NC_024463.2:157118334–157120203(+)	LOC109939952		− 7.05	2.62E−13	3.20E−10	Uncharacterized protein
NC_024466.2:123263353–123266407(−)	LOC100285804		− 8.30	3.63E−13	4.19E−10	SNF1-related protein kinase regulatory subunit beta-1
NC_024461.2:138093686–138100193(−)	LOC103650615		− 6.99	1.04E−12	1.10E−09	Protein SUPPRESSOR OF GENE SILENCING 3 homolog
NC_024467.2:16807555–16809390(−)	LOC103639764		− 7.56	0.000237	0.018867	Chromatin-mediated maintenance of transcription, Transcription elongation factor 1 homolog

Gene ontology analysis showed several DEGs involved in GO terms such as nutrient reservoir activity, molecular carrier activity, detoxification, and immune system process were significantly up-regulated in DMI 81 leaf compared to DMI 56 leaf (Fig. 6). While in DMI 81 root, DEGs involved in symplast and cell junction GO terms were down-regulated while detoxification and immune system GO terms were up-regulated (Fig. 7). Further, genes related to amino-acid synthesis and metabolism (for example; Zm00001d048050, Zm00001d028750) were down-regulated, while genes related to the release of amino acids were up-regulated (for example; Zm00001d009944, Zm00001d031486, Zm00001d016476, and Zm00001d012667) which suggests that during the early stages of N-deficiency, recycling might be the main source of fulfilling the initial demand of N for growth and development of plant^41^.

Previously, many transcription factors (TFs), such as WRKY, MYB, bHLH, bZIP have been reported to play an important role under N-deficiency stress^68,69^. In our study, few members of WRKY and bZIP TF families were found to be up-regulated in the tolerant genotype compared to susceptible one or down-regulated in the susceptible genotype compared to tolerant one. Similar results have been observed in rice where WRKY TFs were up-regulated under N-deficiency stress^23^. Besides, many other members belonging to various TFs such as basic helix loop helix, dehydrin, and late embryogenic abundant TF were found to be down-regulated in the root tissue in the current investigation. These TFs might plays important role in signaling and conferring tolerance against low-N stress in maize. Overall, it was observed that differential expression of genes was more prominent in roots as compared to leaf. This may stem from the fact that roots are generally responsible for nutrient acquisition (such as N) and under a nutrient-deficient scenario, more biological activity, like signal transduction, etc., would occur more in roots. Understanding the differential expression profiles of key genes in the roots of contrasting lines is important in elucidating the genetic determinants of N-deficiency tolerance in maize.

## Conclusion

Comparative transcriptome analysis reveals that adaptive characters like increased root length and decreased shoot biomass under N-deficient conditions are also linked to the dysregulation of various genes involved in different metabolic pathways. Several potential genes were identified which might be involved in determining NUE in maize. The genes encoding for N transporters, enzymes involved in amino acid metabolism, TFs (MYB 36, AP2-EREBP, etc.), and stress-responsive—genes exhibited the genotypic dependent pattern of expression. The present study opens up the scope for the investigation to further examine and elucidate the precise role of highly dysregulated key genes in N-deficiency stress tolerance in maize. Further, the candidate genes identified in the present study may be utilized for molecular marker-assisted breeding towards the development of low-N stress-tolerant maize plants.

## Methods

### Plant material, stress treatment, and RNA extraction

Two maize inbred lines, DMI 56 and DMI 81 were used in this study which was developed by the ICAR-Indian Institute of Maize Research, India. These lines were selected based on a previous study that found DMI 56 as a tolerant line and DMI 81 as a susceptible line for N stress under field conditions^19^. The seeds were surface sterilized with 70% ethanol for 2 min followed by washing with sterile water 4–5 times. Subsequently, the seeds were treated with 0.1% HgCl_2_ for 10 min followed by washing with sterile water four times and allowed to germinate at room temperature in wet germination paper in the dark. After 1 week, germinated seedlings of almost similar length were grown hydroponically in plastic trays for 3 days in Hoagland solution having sufficient N, after which the seedlings were carefully removed and two sets were prepared using the 10-day-old seedlings for the study. One set was transferred to modified Hoagland’s hydroponic solution with sufficient N while the second set was allowed to grow under N-deficient conditions. Plants were grown until symptoms of N-deficiency were observed which was after 21 days in green-house under controlled conditions, i.e. 30/22 (± 2) °C day/night temperatures, 14/10 dark/light hours and 40–50% humidity. The nutrient medium (Hoagland solution) was changed every third day. In the nutrient medium, nitrate and ammonium-containing salt like KNO_3_ (1 M), Ca(NO_3_)·4H_2_O (1 M), NH_4_H_2_PO_4_ (1 M) were replaced by K_2_SO_4_ (1 M), CaCl_2_ (1 M), KH_2_PO_4_ (1 M). Leaf and root samples from DMI 56 and DMI 81 grown hydroponically were collected after 21 days, half of them were used immediately for measuring growth parameters while the remaining were frozen in liquid nitrogen, stored at − 80 °C, and later used for RNA isolation. Total RNA was extracted from 100 mg leaf and root tissues using Spectrum plant total RNA Kit (Sigma Aldrich, USA) as per the manufacturer's instructions. RNA yield and quality were determined using a Nanodrop spectrophotometer (Thermo Scientific, USA).

### Illumina RNA-sequencing

High-throughput RNA sequencing of RNA derived from the root and leaf tissue of tolerant (DMI 56) and susceptible inbred line (DMI 81) was performed on the Illumina platform by NxGenBio Life Sciences, Delhi, India. For the same, eight cDNA libraries were constructed using the leaf and root tissues from contrasting lines. FastQC (version 0.11.5, http://www.bioinformatics.babraham.ac.uk/projects/fastqc/) was used for quality checks for base quality score distribution, sequence quality score distribution, average base content per read, GC distribution in the reads, etc. RNA-seq generated a total of 63.51 GB of high-quality data from eight c-DNA libraries and clean processed reads were aligned to the reference genome i.e. maize B73 version 4 using minimap2. Mapping was carried out using minimap2 software at default parameters with *Zea mays* ref_B73_RefGen_v4 assembly. The quality reads showed 88.10–97.55% mapping percentage to the B73 genome (Supplementary Table 10). Annotation of reference genes was carried out using Uniprot Batch retrieval and Kyoto Encyclopedia of Genes and Genomes (KEGG). Filtered reads are mapped to the genes using bwa 0.7.5 (https://bioweb.pasteur.fr/packages/pack@bwa@0.7.5a) and SAM tools.0.1.19 (https://sourceforge.net/projects/samtools/files/samtools/0.1.19/samtools-0.1.19.tar.bz2) were used to calculate counts mapped with respect to each gene in RNA-Seq data. Later on DESeq R package (https://www.bioconductor.org/packages//2.10/bioc/html/DESeq.html) were used to do differential expression analysis.. The details of samples and various comparisons carried out in the present study are mentioned in Table 2. Mercator was used for annotation and Mapman 3.5.1R2 for filtering differentially expressed genes (DEGs). Significant DEGs were identified based on the statistical significance (*p* value ≤ 0.05) and log2 Fold change (≥ 2 for up-regulated and ≤ − 2 for down-regulated).

### Gene ontology and pathway analysis

Different Public reference databases including National Centre for Biotechnology Information (NCBI) non-redundant (nr), Swiss-Prot and UniProt Reference Clusters (UNIREF) were used to fix unigene identity through Basic Local Alignment Search Tool (BLAST) that has a sequence similarity index of up to an E value < 1.0E−5. Annocript program was employed for Unigene classification and assignment of GO terms to assembled transcripts. Functional categories were based on cellular components, biological processes, and molecular functions. Web Gene Ontology Annotation Plot (WEGO) tool was used for constructing GO plots representing sorted transcripts. Enrichment analysis of the top 20 DEGs was conducted. The Cluster of Orthologous Groups (COG) database was employed to achieve operational cataloging of unigenes and their role in different metabolic pathways was deduced through the KEGG database. DEGs of different comparison assemblies were represented employing numerous plots like HeatMap, Circos, and MapMan (v3.51R2).

### Quantitative real-time PCR (qRT-PCR) analysis

Total RNA was isolated from frozen leaf and root samples using Spectrum™ Plant Total RNA Kit™ (Sigma) according to the manufacturer’s protocol and stored at − 80 °C. The quality and concentration of the isolated RNA were assessed by a NanoDrop spectrophotometer (Nano 200). The cDNA was synthesized from total RNA using (Takara Bio) as per manufacturer protocol and stored at − 20 °C. The coding sequence of selected DEGs was obtained from NCBI and gene-specific qRT-PCR primers were designed using IDT Primer designer and cross-checked by NCBI Primer-BLAST. The list of primers is presented in Supplementary Table S11. The qRT-PCR was performed in triplicates using the real-time PCR (Agilent Technologies, USA) detection system as described elsewhere^70^. The qRT-PCR of selected genes was carried out in those comparisons only (out of six total comparisons) in which the particular gene has significant expression in RNA-Seq analysis. The PCR program was set for 40 cycles. The maize *Actin* gene was used as an internal control to normalize gene expressions. Melting curves were analyzed and the relative fold change (log2 scale) in gene expression was calculated using the 2^−ΔΔCt^ method^71^.

### Plant ethics statements

All the methods used in the current study were carried out following relevant guidelines and regulations.

All experimental protocols were approved by a named institutional and/or licensing committee.

## Supplementary Information

Supplementary Information 1.

Supplementary Information 2.

## Data Availability

The RNA-sequence data has been deposited in the NCBI database. The submission details are: Bioproject PRJNA731645; SRA Accession nos. SUB9657581; and BioSamples: SAMN19292165, SAMN19292166, SAMN19292167, SAMN19292168, SAMN19292169, SAMN19292170, SAMN19292171, SAMN19292172.
