# Supplementary material for: Global gene expression profiling under nitrogen stress identifies key genes involved in nitrogen stress adaptation in maize (Zea mays L.)
Source: Sci Rep. 2022 Mar 10;12:4211. doi: 10.1038/s41598-022-07709-z (PMC8913646; doi:10.1038/s41598-022-07709-z)
Supplement: Supplementary file 2 — Supplementary Information 2. [file 41598_2022_7709_MOESM2_ESM.pdf]

**Global gene expression profiling under nitrogen stress identifies key genes involved in nitrogen stress adaptation in maize (*Zea mays L.*)**

*Prabha Singh[1,2,3*], Krishan Kumar[1*], Abhishek Kumar Jha[1], Pranjal Yadava[2], Madan Pal[2], Sujay Rakshit[1] and Ishwar Singh[1#]*

**Krishan Kumar contributed equally and considered as co-first author.*

*[#]Corresponding author [E-mail: ishwar.singh@icar.gov.in]*

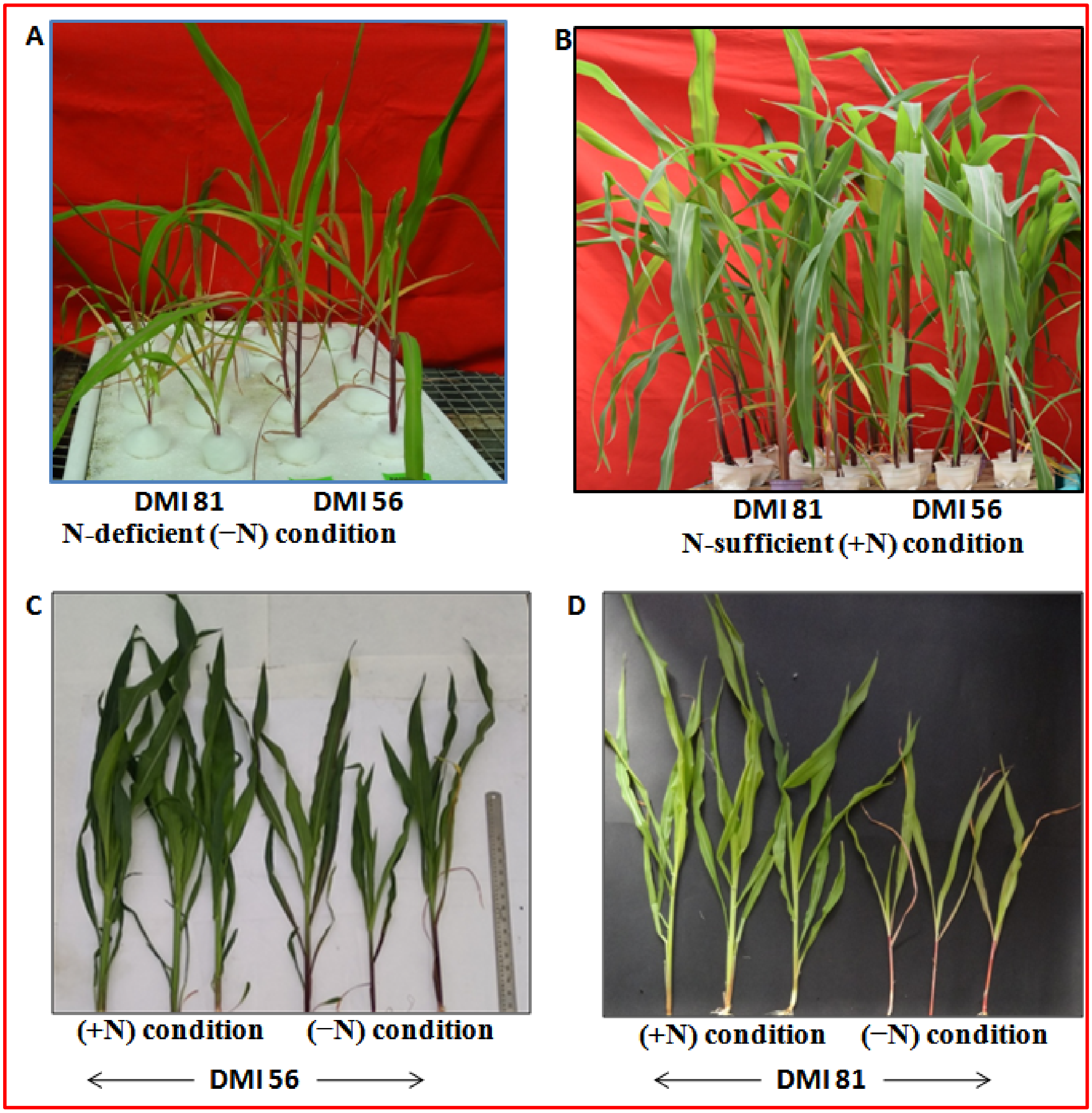


**Supplementary Fig S1.** Effect of N-stress on two contrasting maize inbred lines DMI 56 (tolerance to nitrogen stress) and DMI 81 (susceptible to nitrogen stress). Plants were grown hydroponically under N-deficient **(A)** and sufficient/control **(B)** conditions for 21 days. DMI 56 **(C)** and DMI 81 **(D)** lines under N-sufficient and -deficient conditions.

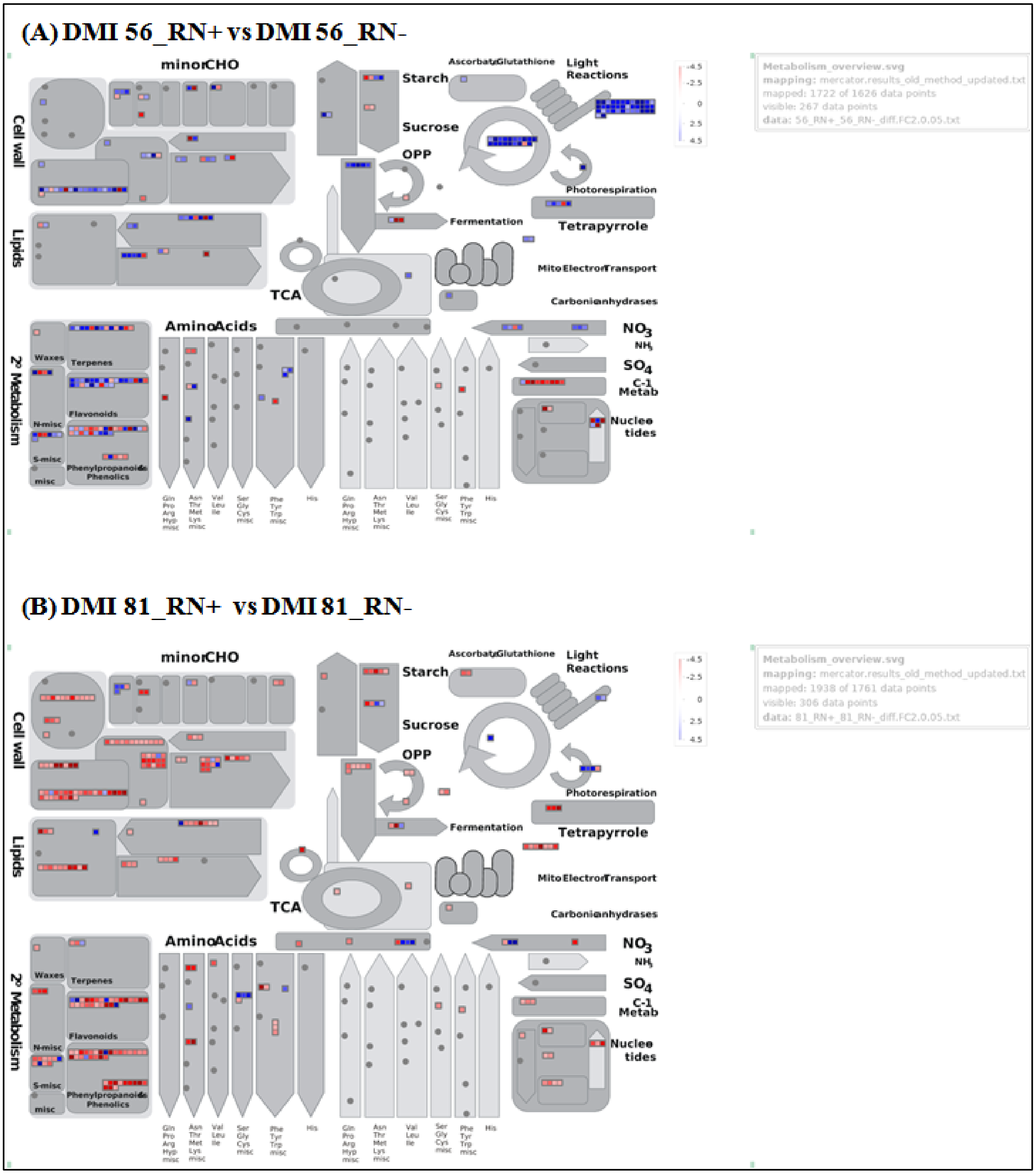


**Supplemental Fig S2:** MapMan-based visualization of the DEGs in root tissues of **(A)** DMI 56 (tolerant to low-N stress) and **(B)** DMI 81 (susceptible to low-N stress) under low-nitrogen stress compared to their respective control (sufficient-N sample). Small blue and red colour squares represent up-and down-regulated genes, respectively at the amplitude of 4.5 to −4.5 (log2-value). Letters R, N+, and N- represent root, sufficient nitrogen, and nitrogen deficiency conditions, respectively.

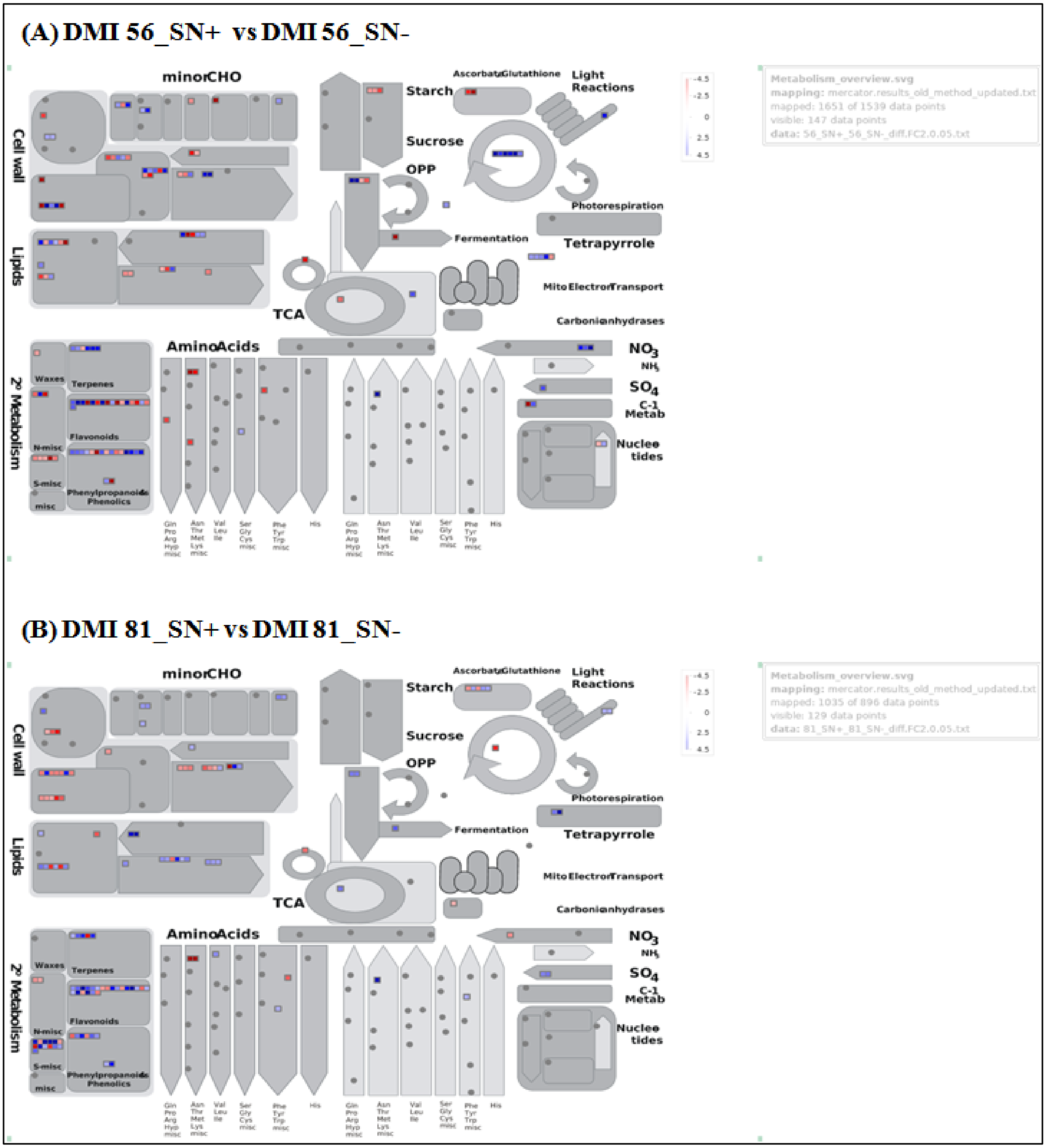


**Supplemental Fig S3:** MapMan-based visualization of the DEGs in leaf tissues of **(A)** DMI 56 (tolerant to low-N stress) and **(B)** DMI 81 (susceptible to low-N stress) under low-nitrogen stress as compared to their respective control (sufficient-N sample). Small blue and red colour squares represents up- and down-regulated genes, respectively at the amplitude of 4.5 to −4.5 (log2-value). Letters S, N+, and N- represent leaf, sufficient nitrogen, and nitrogen deficiency conditions, respectively.

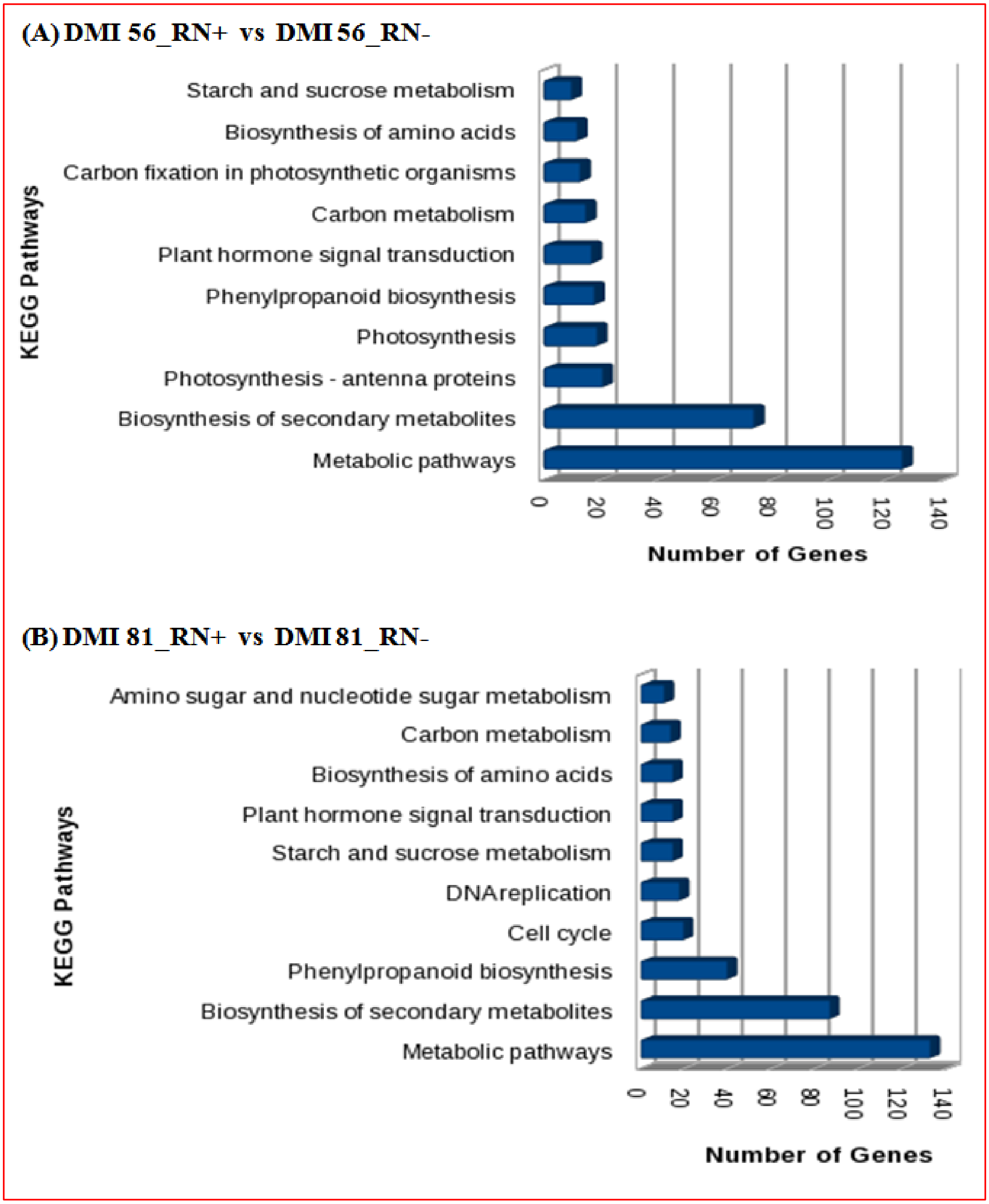


**Supplemental Fig S4:** KEGG pathway enrichment analysis of DEGs in comparison **(A)** (DMI 56_RN+ vs DMI 56_RN-) **(B)** (DMI 81_RN+ vs DMI 81_RN-). The top 10 pathways from the KEGG enrichment analysis are shown as a bar chart. The terms of the KEGG pathways are depicted on the y-axis. The no of DEGs is shown as the length of the histogram. Letters R, N+, and N- represent root, nitrogen-sufficient, and nitrogen- deficiency conditions, respectively.

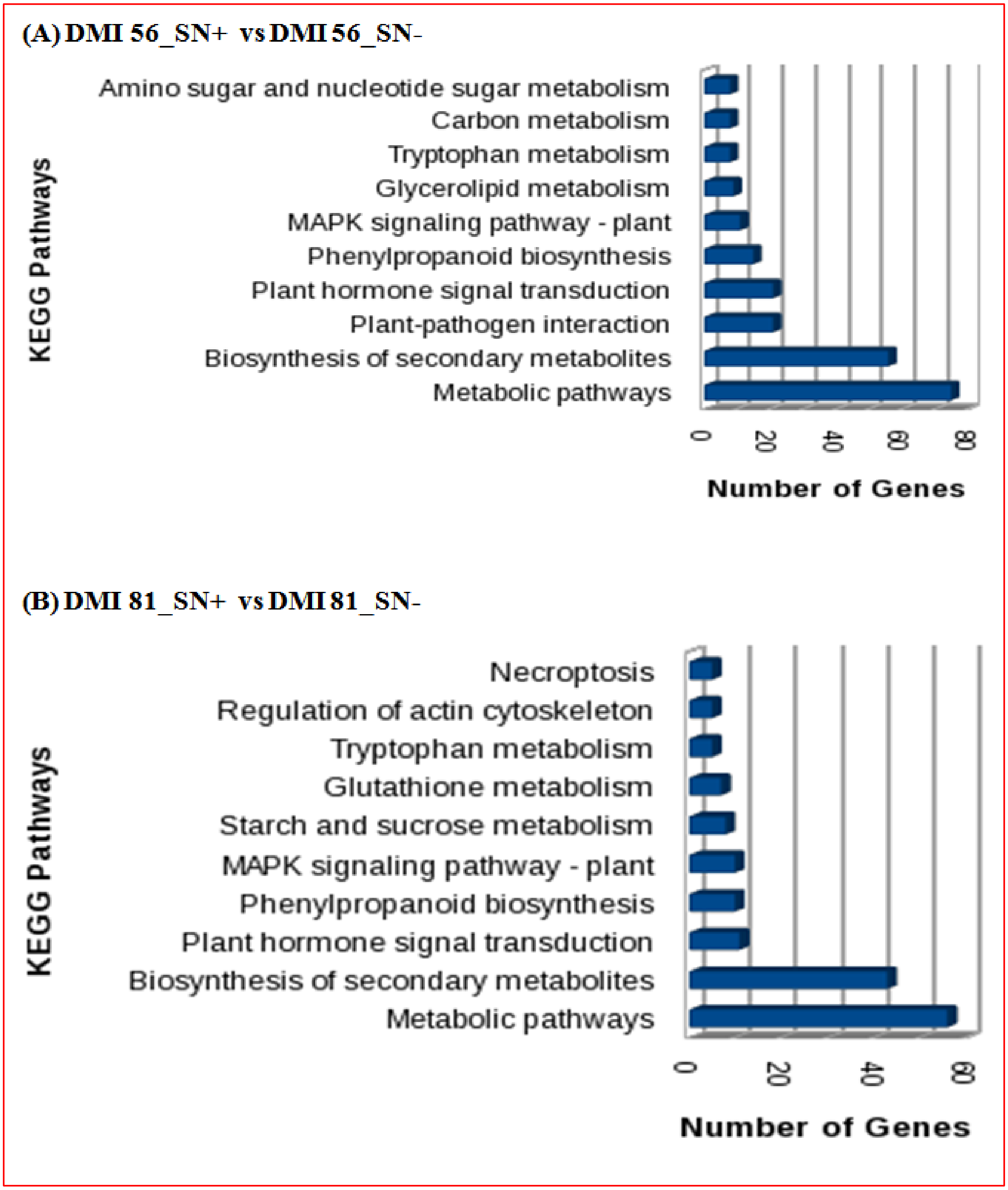


**Supplemental Fig S5:** KEGG pathway enrichment analysis of DEGs in comparison **(A)** (DMI 56_SN+ vs DMI 56_SN-) **(B)** (DMI 81_SN+ vs DMI 81_SN-). The top 10 pathways from the KEGG enrichment analysis are shown as a bar chart. The terms of the KEGG pathways are depicted on the y-axis. The no of DEGs is shown as the length of the histogram. Letter S, N+, and N- represents leaf, nitrogen-sufficient, and nitrogen- deficiency conditions, respectively.

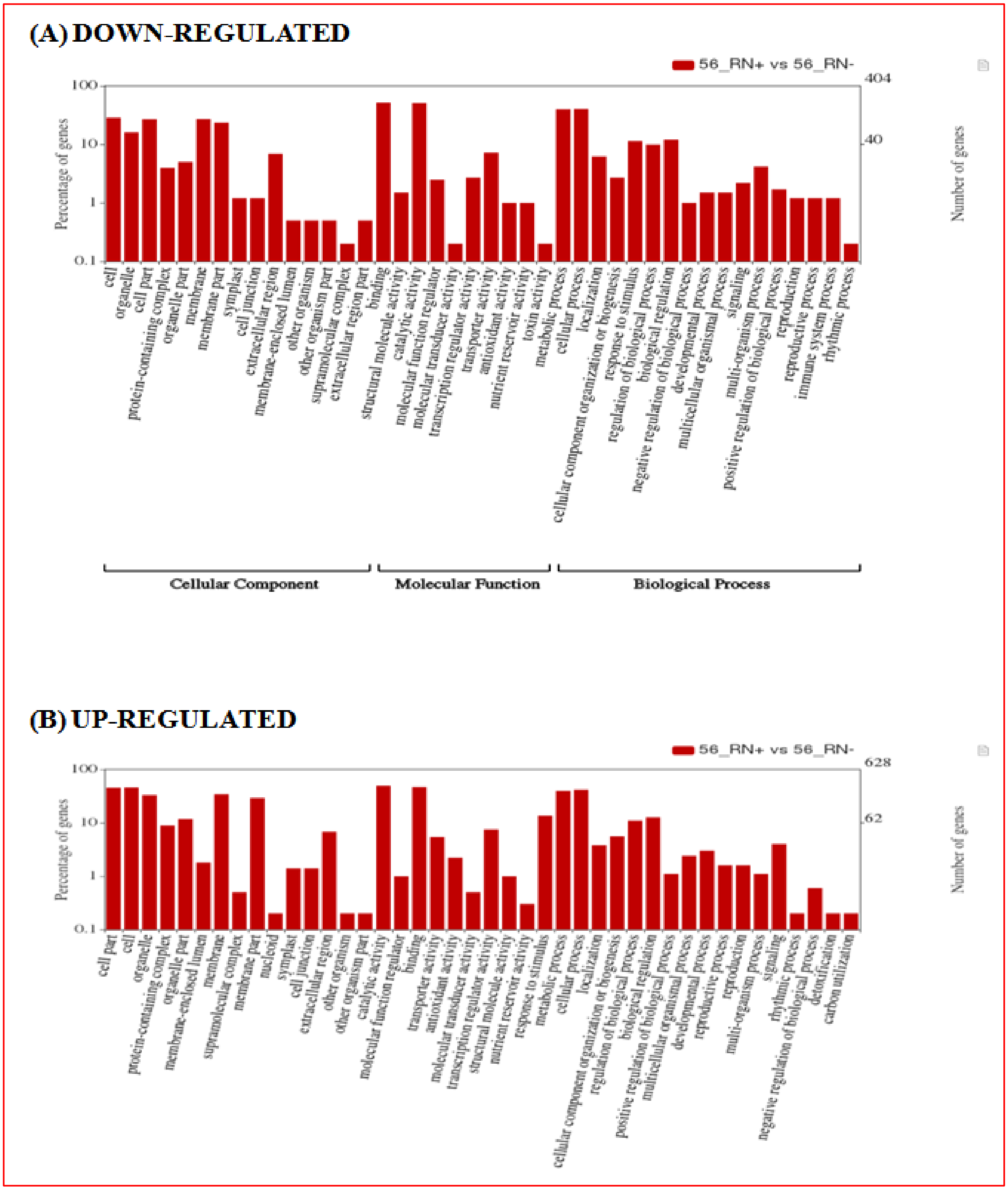


**Supplemental Fig S6:** Wego plot for **(A)** down-regulated and **(B)** up-regulated GO classification in accordance to GO groups: molecular function, biological process and cellular component in DMI 56 root under low-N stress compared to its control (sufficient N) (named as DMI 56_RN+ vs DMI 56_RN-).

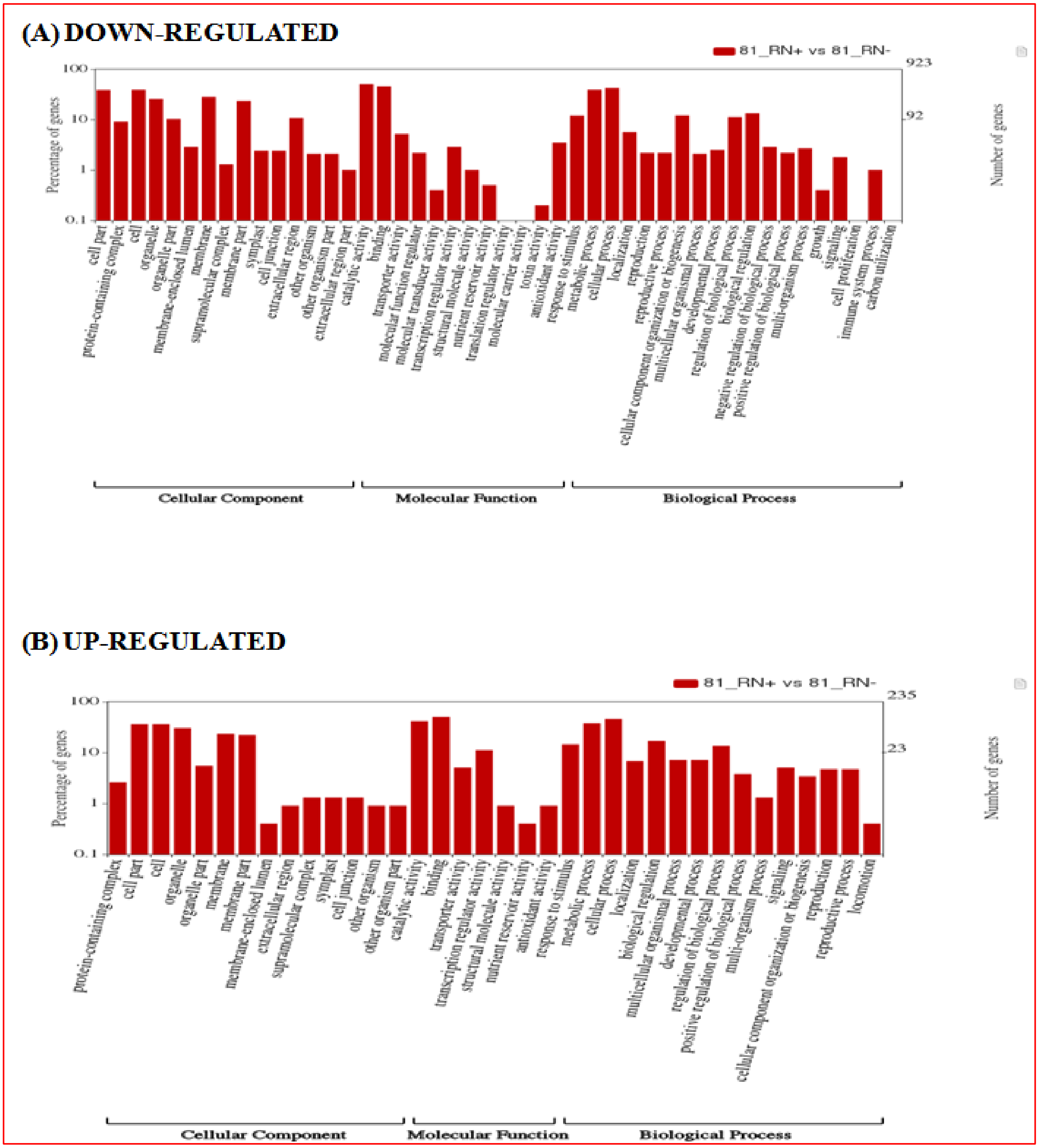


**Supplemental Fig S7:** Wego plot for **(A)** down-regulated and **(B)** up-regulated GO classification in accordance to GO groups: molecular function, biological process and cellular component in DMI 81 root under low-N stress compared to its control (sufficient N) (named as DMI 81_RN+ vs DMI81_RN-).

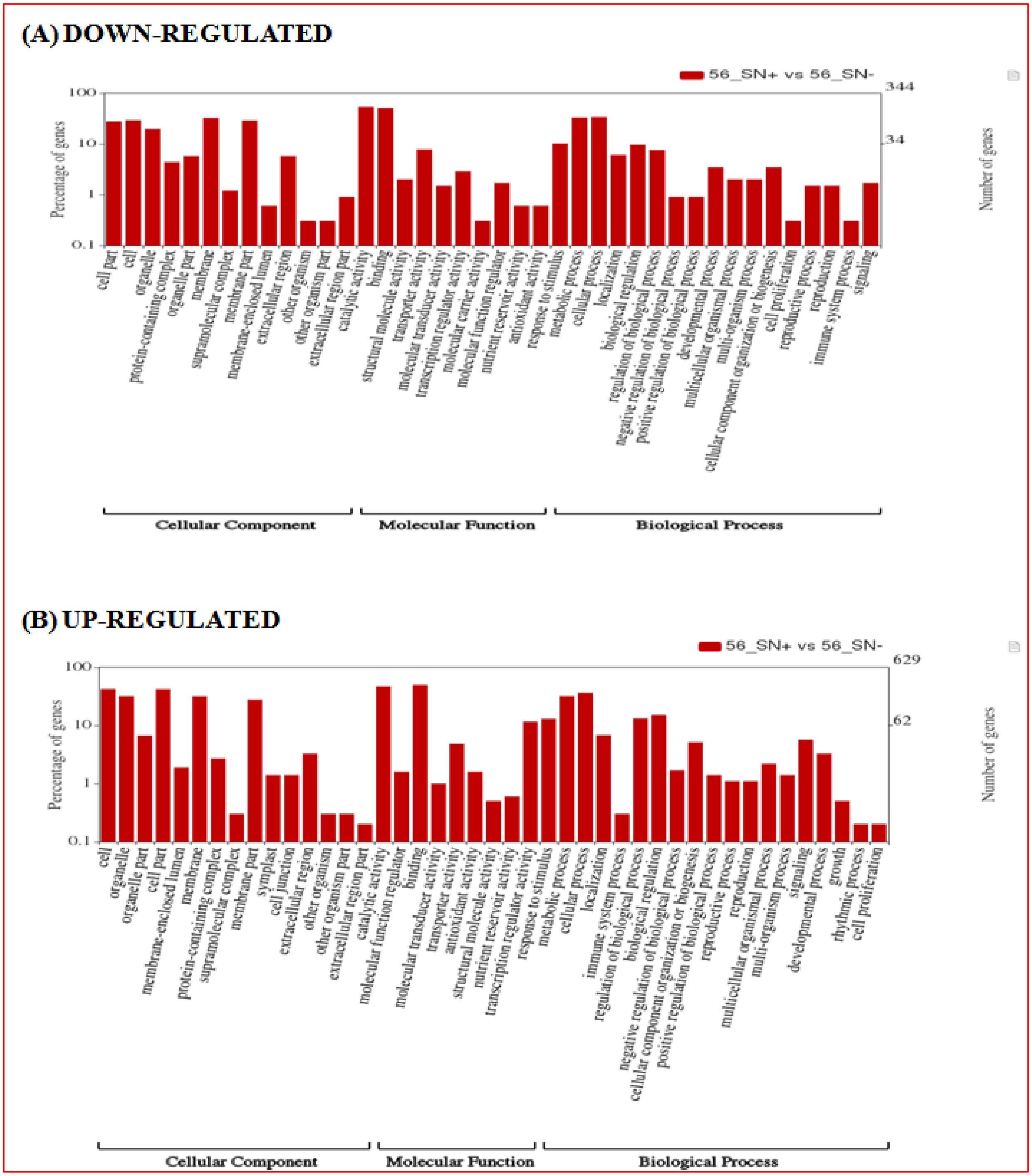


**Supplemental Fig S8:** Wego plot for **(A)** down-regulated and **(B)** up-regulated GO classification in accordance to GO groups: molecular function, biological process and cellular component in DMI 56 leaf under low-N stress compared to its control (sufficient N) (named as DMI 56_SN+ vs DMI56_SN).

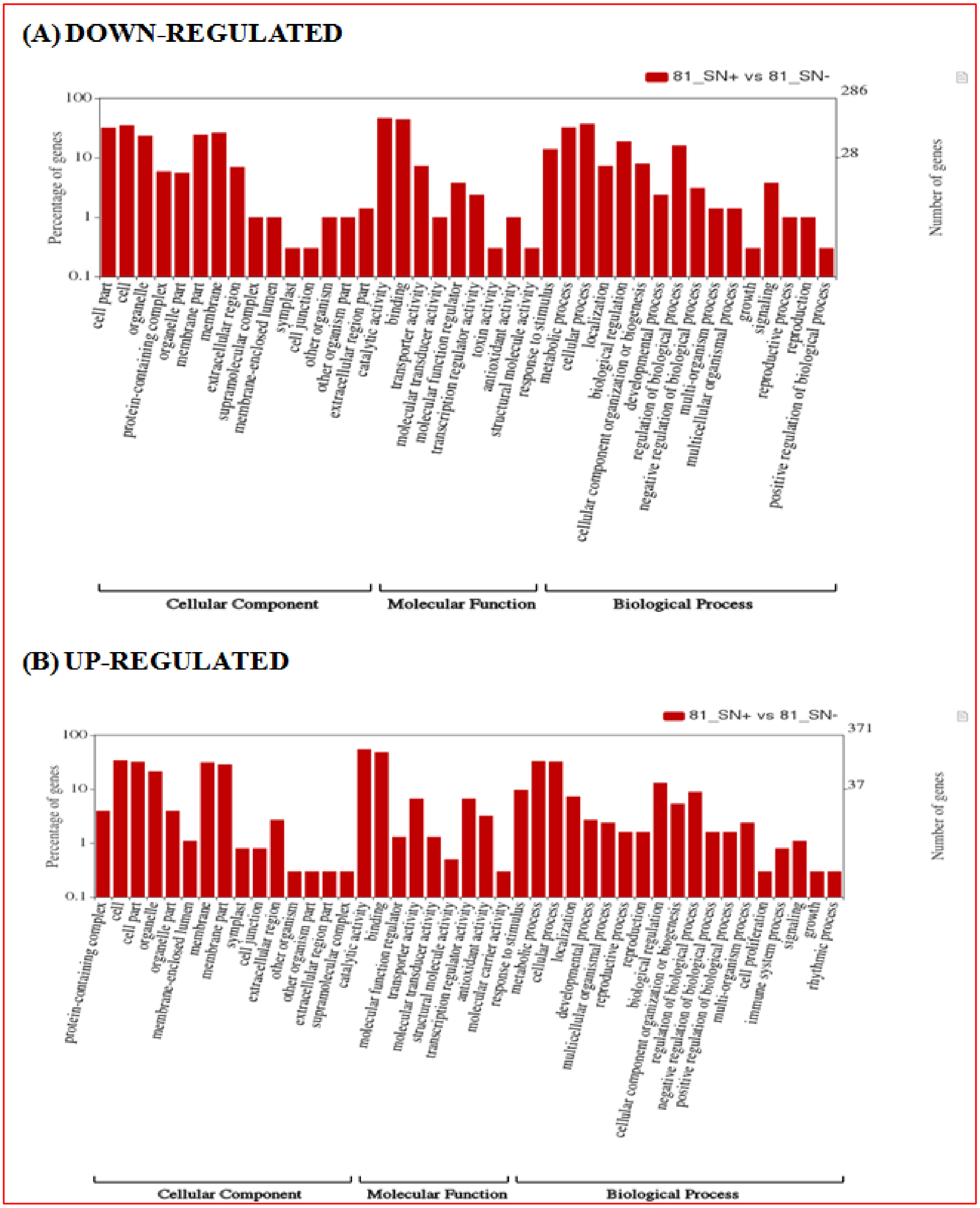


**Supplemental Fig S9:** Wego plot for (A) down-regulated and (B) up-regulated GO classification in accordance to GO groups: molecular function, biological process and cellular component in DMI 81 leaf under low-N stress compared to its control (sufficient N) (named as DMI 81_SN+ vs DMI81_SN-).

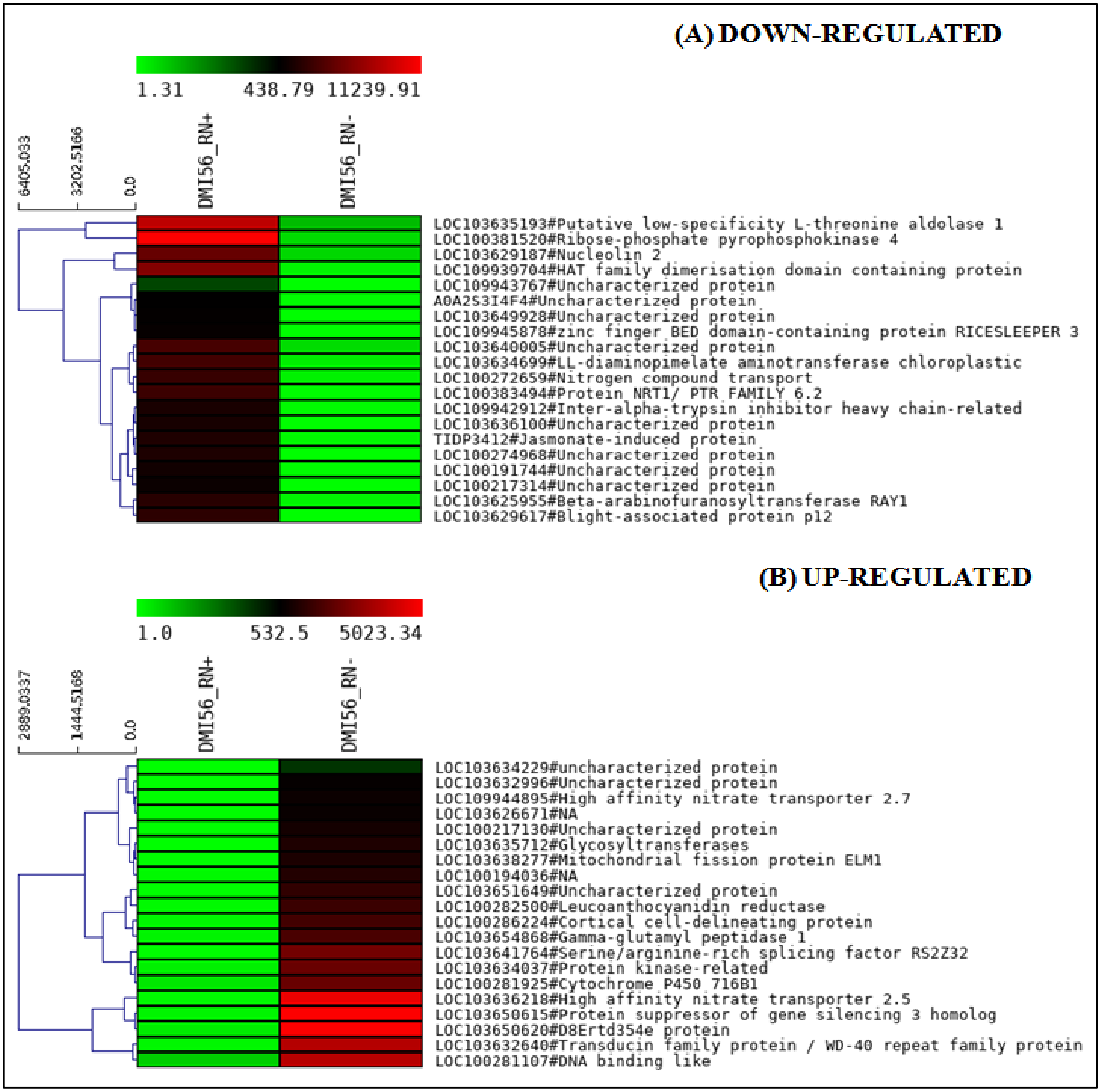


**Supplemental Fig S10:** Heat map depicting top 20 down-regulated **(A)** and up-regulated **(B)** genes with *p*-value < 0.05 in DMI 56 root under low-N stress compared to its control (sufficient N) (named as DMI 56_RN+ vs DMI 56_RN-). The plants were grown in hydroponic culture with low N and sufficient N supply. In the heat maps, each horizontal line refers to a gene. Relatively up-regulated genes are shown in red colour, whereas down-regulated genes are shown in green colour.

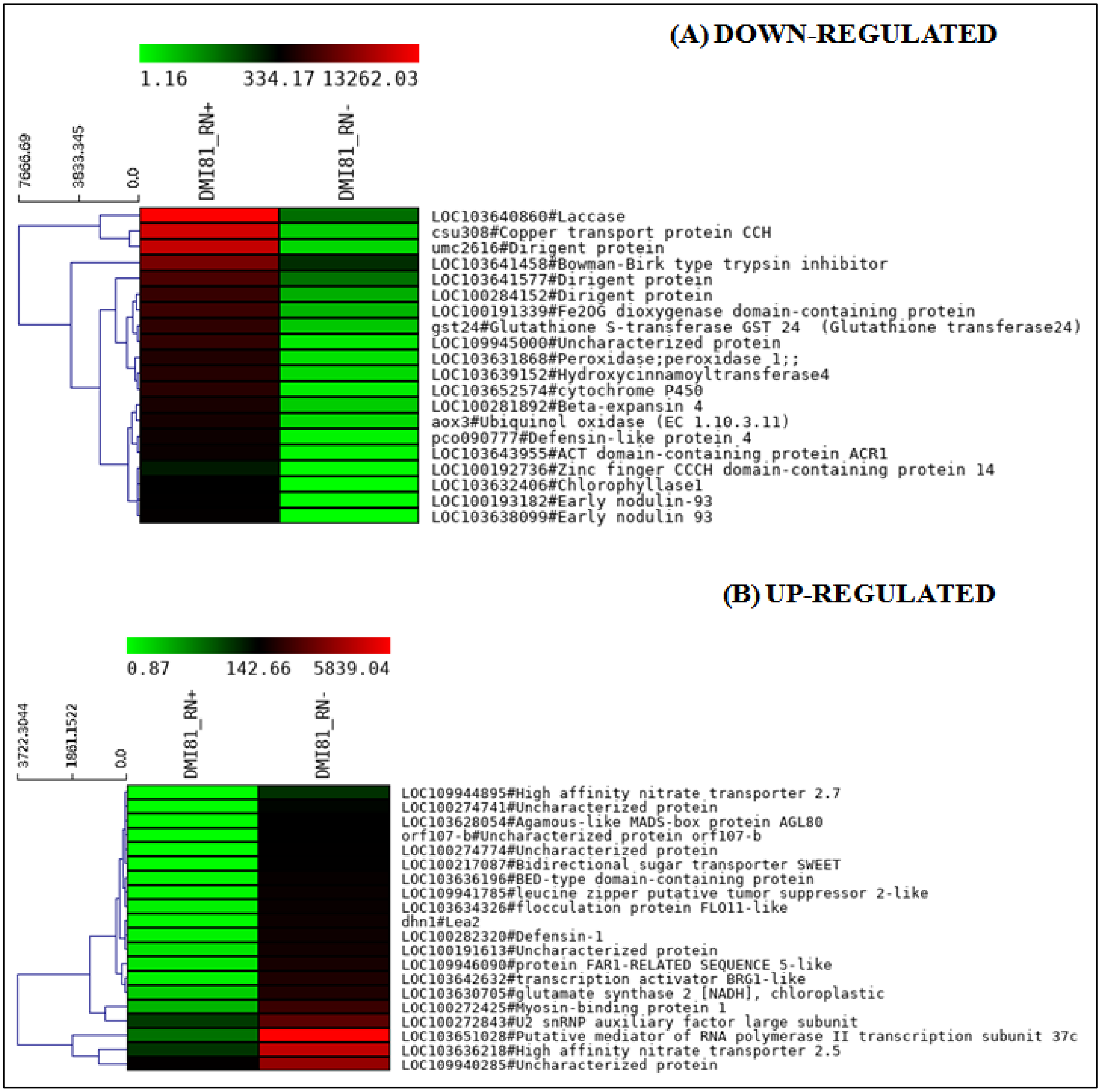


**Supplemental Fig S11:** Heat map depicting top 20 down-regulated **(A)** and up-regulated **(B)** genes with *p*-value < 0.05 in DMI 81 root under low-N stress compared to its control (sufficient N) (named as DMI 81_RN+ vs DMI 81_RN-). The plants were grown in hydroponic culture with low N and sufficient N supply. In the heat maps, each horizontal line refers to a gene. Relatively up-regulated genes are shown in red colour, whereas down-regulated genes are shown in green colour.

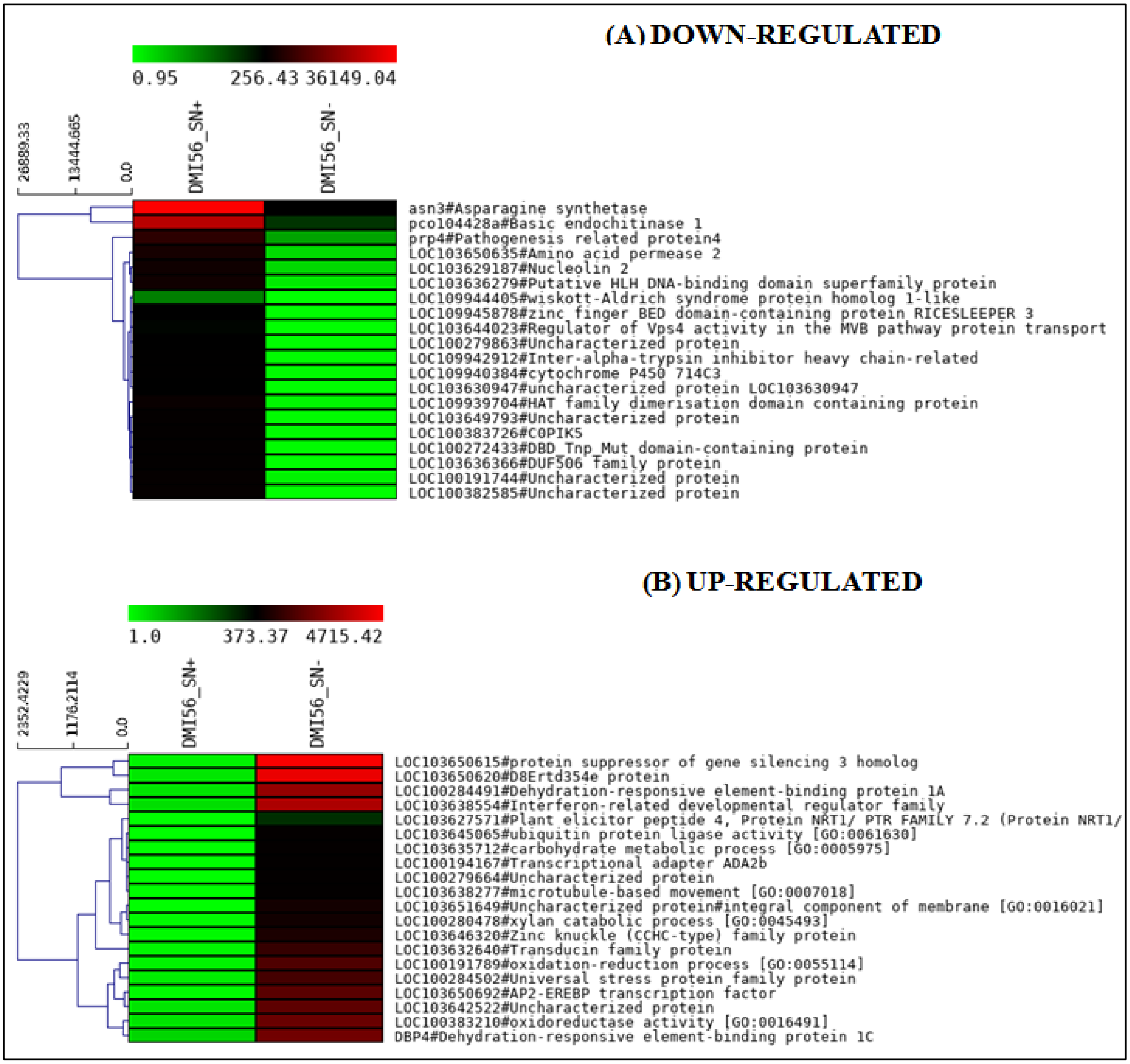


**Supplemental Fig S12:** Heat map depicting top 20 down-regulated **(A)** and up-regulated **(B)** genes with *p*-value < 0.05 in DMI 56 leaf under low-N stress compared to its control (sufficient N) (named as DMI 56_SN+ vs DMI 56_SN-). The plants were grown in hydroponic culture with low N and sufficient N supply. In the heat maps, each horizontal line refers to a gene. Relatively up-regulated genes are shown in red colour, whereas down-regulated genes are shown in green colour.

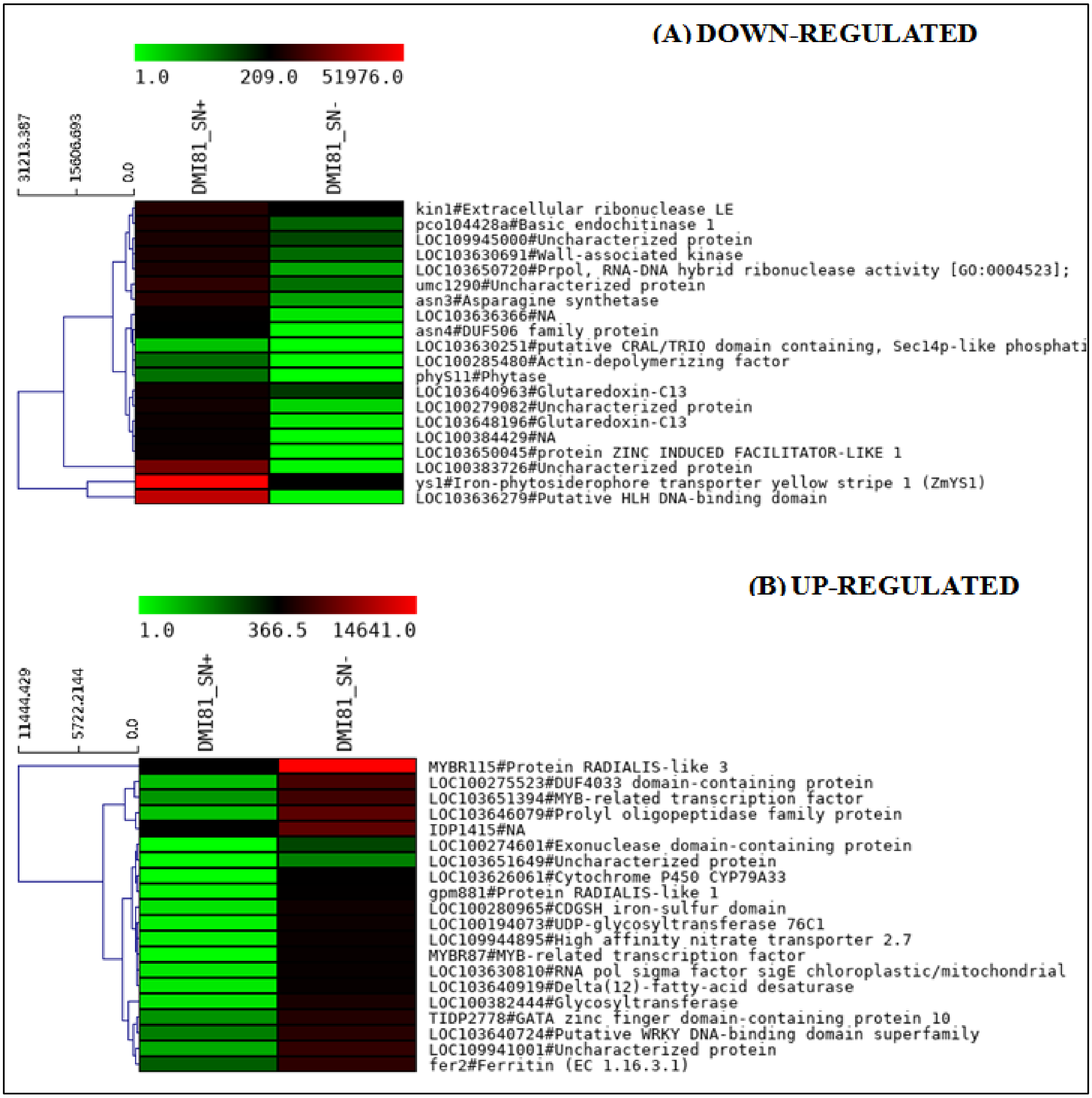


**Supplemental Fig S13:** Heat map depicting top 20 down-regulated **(A)** and up-regulated **(B)** genes with *p*-value < 0.05 in DMI 81 leaf under low-N stress compared to its control (sufficient N) (named as DMI 81_SN+ vs DMI 81_SN-). The plants were grown in hydroponic culture with low N and sufficient N supply. In the heat maps, each horizontal line refers to a gene. Relatively up-regulated genes are shown in red colour, whereas down-regulated genes are shown in green colour.

**Supplementary Table S2:** Selected top 20 up-regulated genes in DMI 56 root under low-N stress compared to its control (sufficient N) (named as DMI 56_RN+ vs DMI 56_RN-). Letters R, N+ and N- represent root, sufficient nitrogen, and nitrogen-deficiency conditions, respectively.

| **DMI 56_RN+ vs DMI 56_RN-** | | | | | |
|---|---|---|---|---|---|
| **ID** | **Gene I'D** | **$Log_2$ fold change** | **P value** | **FDR** | **Gene Description** |
| NC_024466.2:158415465-158417502(+) | LOC103636218 | 8.41963 | 7.90E-19 | 2.43E-14 | High-affinity nitrate transporter 2.5 |
| NC_024461.2:138093686-138100193(-) | LOC103650615 | 7.930844 | 9.84E-18 | 8.24E-14 | Protein suppressor of gene silencing 3 homolog |
| NC_024468.2:126118262-126120658(+) | LOC103641764 | 8.547843 | 1.47E-17 | 1.02E-13 | Serine/arginine-rich splicing factor RS2Z32 |
| NC_024461.2:232951331-232954396(-) | LOC103651649 | Inf | 2.72E-17 | 1.62E-13 | Uncharacterized protein |
| NC_024465.2:101004710-101009817(-) | LOC103632640 | 7.863483 | 3.19E-17 | 1.67E-13 | Transducin family protein / WD-40 repeat family protein |
| NC_024461.2:138805064-138814004(+) | LOC103650620 | 7.041498 | 1.72E-15 | 5.53E-12 | D8Ertd354e protein |
| NC_024466.2:113319211-113321231(-) | LOC103635712 | Inf | 2.85E-15 | 8.53E-12 | Glycosyltransferases |
| NC_024460.2:195290931-195293498(+) | LOC100217130 | Inf | 5.44E-14 | 1.27E-10 | Uncharacterized protein |
| NC_024467.2:37432851-37435462(+) | LOC103638277 | 9.411959 | 2.16E-13 | 4.53E-10 | Mitochondrial fission protein ELM1 |
| NC_024468.2:144452238-144454289(-) | LOC100282500 | 7.314584 | 4.79E-13 | 9.12E-10 | Leucoanthocyanidin reductase |
| NC_024465.2:98116798-98117776(-) | LOC103634037 | 6.252393 | 2.06E-12 | 3.31E-09 | Protein kinase-related |
| NC_024462.2:156545940-156546701(+) | LOC100286224 | 6.61879 | 4.18E-12 | 6.04E-09 | Cortical cell-delineating protein |

| | | | | | |
|---|---|---|---|---|---|
| NC_024465.2:140065077-140066304(-) | LOC103632996 | Inf | 1.05E-11 | 1.29E-08 | Uncharacterized protein |
| NC_024462.2:240079626-240080879(-) | LOC103654868 | 6.284761 | 1.16E-11 | 1.38E-08 | Gamma-glutamyl peptidase 1 |
| NC_024462.2:5969504-5971912(+) | LOC100281925 | 5.574259 | 8.09E-11 | 8.91E-08 | Cytochrome P450 716B1 |
| NC_024461.2:230035429-230038824(-) | LOC109944895 | 8.347387 | 1.02E-10 | 1.10E-07 | High-affinity nitrate transporter 2.7 |
| NC_024468.2:106318814-106321232(-) | LOC100281107 | 5.201142 | 1.69E-10 | 1.68E-07 | DNA binding like |
| NC_024465.2:101003482-101007474(+) | LOC100194036 | 6.476499 | 3.12E-10 | 2.97E-07 | NA |
| NC_024463.2:88008903-88012287(-) | LOC103626671 | 7.563962 | 8.17E-10 | 7.28E-07 | NA |
| NC_024465.2:140066671-140067924(-) | LOC103634229 | Inf | 1.13E-09 | 9.69E-07 | uncharacterized protein |

**Supplementary Table S3:** Selected top 20 down-regulated genes in DMI 56 root under low-N stress compared to its control (sufficient N) (named as DMI 56_RN+ vs DMI 56_RN-).

| **DMI 56_RN+ vs DMI 56_RN-** | | | | | |
|---|---|---|---|---|---|
| **ID** | **Gene I'D** | **$Log_2$ fold change** | **P value** | **FDR** | **Gene Description** |
| NC_024463.2:72705848-72708598(-) | LOC109939704 | -8.47587 | 1.16E-18 | 2.43E-14 | HAT family dimerisation domain-containing protein |
| NC_024466.2:62579423-62581864(-) | LOC100383494 | -9.15771 | 2.46E-18 | 3.43E-14 | Protein NRT1/ PTR FAMILY 6.2 |
| NC_024464.2:91462875-91463803(+) | LOC103629617 | -9.81172 | 7.20E-18 | 7.54E-14 | Blight-associated protein p12 |
| NC_024462.2:181681444-181682786(-) | LOC109945878 | -8.17014 | 6.05E-10 | 5.63E-07 | zinc finger BED domain-containing protein RICESLEEPER 3 |

| | | | | | |
|---|---|---|---|---|---|
| NC_024461.2:32393407-32394800(-) | LOC103649928 | -8.01991 | 2.17E-09 | 1.69E-06 | Uncharacterized protein |
| NC_024462.2:165271169-165273576(+) | LOC100381520 | -7.51045 | 1.29E-15 | 4.91E-12 | Ribose-phosphate pyrophosphokinase 4 |
| NC_024461.2:54139202-54139903(+) | LOC100274968 | -9.36206 | 1.52E-15 | 5.31E-12 | Uncharacterized protein |
| NC_024466.2:149851012-149852859(-) | LOC103636100 | -9.14369 | 2.10E-14 | 5.87E-11 | Uncharacterized protein |
| NC_024466.2:5875118-5881172(+) | LOC103634699 | -6.83244 | 2.66E-14 | 6.97E-11 | LL-diaminopimelate aminotransferase chloroplastic |
| NC_024464.2:10069849-10071772(+) | LOC100272659 | -6.89883 | 4.21E-14 | 1.04E-10 | Nitrogen compound transport |
| NC_024462.2:198407157-198409508(-) | A0A2S3I4F4 | -7.73507 | 1.91E-08 | 1.08E-05 | Uncharacterized protein |
| NC_024463.2:11855624-11859798(-) | LOC103625955 | -7.09084 | 1.44E-13 | 3.16E-10 | Beta-arabinofuranosyltransferase RAY1 |
| NC_024468.2:122393248-122395555(-) | LOC109942912 | -7.64506 | 4.63E-13 | 9.12E-10 | Inter-alpha-trypsin inhibitor heavy chain-related |
| NC_024464.2:28928493-28931803(+) | LOC103629187 | -6.11678 | 5.63E-13 | 1.02E-09 | Nucleolin 2 |
| NC_024459.2:63317828-63324872(+) | LOC103635193 | -6.18538 | 7.45E-13 | 1.30E-09 | Putative low-specificity L-threonine aldolase 1 |
| NC_024461.2:132539492-132540473(-) | TIDP3412 | -7.0791 | 9.67E-13 | 1.62E-09 | Jasmonate-induced protein |
| NC_024462.2:196029366-196032725(-) | LOC100217314 | -9.55279 | 3.70E-12 | 5.59E-09 | Uncharacterized protein |
| NC_024459.2:249828810-249830061(+) | LOC109943767 | -7.92476 | 8.50E-07 | 0.000254 | Uncharacterized protein |
| NC_024461.2:231817612-231818726(+) | LOC100191744 | -7.87561 | 4.40E-12 | 6.15E-09 | Uncharacterized protein |

| | | | | | |
|---|---|---|---|---|---|
| NC_024467.2:66116173-66116535(-) | LOC103640005 | -5.8496 | 4.91E-12 | 6.63E-09 | Uncharacterized protein |

**Supplementary Table S4:** Selected top 20 up-regulated genes in DMI 81 root under low-N stress compared to its control (sufficient N) (named as DMI 81_RN+ vs DMI 81_RN-).

| **DMI 81_RN+ vs DMI 81_RN-** | | | | | |
|---|---|---|---|---|---|
| **ID** | **Gene I'D** | **$Log_2$ fold change** | **P value** | **FDR** | **Gene Description** |
| NC_024461.2:184004253-184007904(-) | LOC103651028 | 6.195975 | 1.52E-10 | 1.26E-06 | Putative mediator of RNA polymerase II transcription subunit 37c |
| NC_024464.2:141324558-141325543(-) | dhn1 | Inf | 3.38E-09 | 1.99E-05 | Lea2 |
| NC_024466.2:158415465-158417502(+) | LOC103636218 | 5.295507 | 7.57E-09 | 3.13E-05 | High affinity nitrate transporter 2.5 |
| NC_024468.2:92359187-92360695(-) | LOC103642632 | 6.579942 | 4.13E-08 | 9.49E-05 | transcription activator BRG1-like |
| NC_024459.2:244884706-244894283(-) | LOC100272425 | 5.095952 | 2.47E-07 | 0.000463 | Myosin-binding protein 1 |
| NC_024460.2:231129791-231130667(+) | LOC100274774 | Inf | 2.93E-07 | 0.000527 | Uncharacterized protein |
| NC_024460.2:236537682-236544091(-) | LOC100272843 | 4.309235 | 1.38E-06 | 0.00178 | U2 snRNP auxiliary factor large subunit |
| NC_024463.2:20265968-20266894(-) | LOC103628054 | Inf | 1.56E-06 | 0.00189 | Agamous-like MADS-box protein AGL80 |
| NC_024468.2:110200453-110201623(+) | LOC100274741 | Inf | 1.91E-06 | 0.002085 | Uncharacterized protein |
| NC_024463.2:192005501-192006241(+) | LOC100282320 | 5.932617 | 2.54E-06 | 0.002585 | Defensin-1 |
| NC_024466.2:68841087-68842987(+) | LOC109941785 | 6.327733 | 2.58E-06 | 0.002585 | leucine zipper putative tumor suppressor 2-like |

| | | | | | |
|---|---|---|---|---|---|
| NC_024462.2:208046149-208049422(-) | LOC109946090 | 5.428511 | 2.92E-06 | 0.002682 | protein FAR1-RELATED SEQUENCE 5-like |
| NC_024464.2:14454177-14465023(-) | LOC109940285 | 3.922351 | 4.61E-06 | 0.003963 | Uncharacterized protein |
| NC_024461.2:230035429-230038824(-) | LOC109944895 | Inf | 5.00E-06 | 0.004133 | High affinity nitrate transporter 2.7 |
| NC_024464.2:167441703-167453268(+) | LOC103630705 | 4.906922 | 5.26E-06 | 0.004211 | glutamate synthase 2 [NADH], chloroplastic |
| NC_024459.2:59948045-59951123(-) | LOC100217087 | 6.791081 | 5.95E-06 | 0.004391 | Bidirectional sugar transporter SWEET |
| NC_007982.1:372870-373193(-) | orf107-b | 7.404726 | 7.31E-06 | 0.004953 | Uncharacterized protein orf107-b |
| NC_024466.2:156961406-156962581(+) | LOC103636196 | 5.847887 | 9.71E-06 | 0.006469 | BED-type domain-containing protein |
| NC_024459.2:227680106-227684609(+) | LOC100191613 | 5.161468 | 1.32E-05 | 0.008281 | Uncharacterized protein |
| NC_024465.2:161004438-161005368(+) | LOC103634326 | 5.522241 | 1.34E-05 | 0.008281 | flocculation protein FLO11-like |

**Supplementary Table S5:** Selected top 20 down-regulated genes in DMI 81 root under low-N stress compared to its control (sufficient N) (named as DMI 81_RN+ vs DMI 81_RN-).

| **DMI 81_RN+ vs DMI 81_RN-** | | | | | |
|---|---|---|---|---|---|
| **ID** | **Gene I'D** | **$Log_2$ fold change** | **P Value** | **FDR** | **Gene Description** |
| NC_024463.2:149398951-149400833(+) | umc2616 | -7.63864 | 3.05E-12 | 1.26E-07 | Dirigent protein |
| NC_024463.2:172644130-172645527(+) | csu308 | -7.3892 | 1.31E-11 | 2.71E-07 | Copper transport protein CCH |

| | | | | | |
|---|---|---|---|---|---|
| | | 8 | | | |
| NC_024461.2:58127334-58128164(-) | LOC109945000 | -6.57352 | 2.78E-11 | 3.23E-07 | Uncharacterized protein |
| NC_024461.2:220486444-220488718 | - | -6.70342 | 3.13E-11 | 3.23E-07 | cytochrome P450 |
| NC_024465.2:1175473-1177144(+) | LOC103631868 | -5.73555 | 4.09E-09 | 2.11E-05 | Peroxidase;peroxidase 1;; |
| NC_024459.2:142569164-142571276(-) | LOC103639152 | -5.53871 | 6.02E-09 | 2.76E-05 | Hydroxycinnamoyltransferase4 |
| NC_024468.2:12275223-12278091(-) | LOC103640860 | -6.11195 | 8.35E-09 | 3.14E-05 | Laccase |
| NC_024467.2:20480643-20481654(+) | LOC103638099 | -8.95465 | 1.06E-08 | 3.65E-05 | Early nodulin 93 |
| NC_024463.2:216862611-216863602(-) | gst24 | -5.22149 | 1.38E-08 | 4.38E-05 | Glutathione S-transferase GST 24 (Glutathione transferase24) |
| NC_024461.2:135359547-135361124(-) | LOC100191339 | -5.13039 | 1.69E-08 | 4.98E-05 | $Fe_2$OG dioxygenase domain-containing protein |
| NC_024459.2:277214406-277217329(+) | LOC103643955 | -6.65944 | 2.22E-08 | 6.10E-05 | ACT domain-containing protein ACR1 |
| NC_024468.2:132238491-132239276(+) | pco090777 | -6.17584 | 3.37E-08 | 8.20E-05 | Defensin-like protein 4 |
| NC_024459.2:187013824-187015222(+) | LOC100284152 | -4.87698 | 5.80E-08 | 0.000123 | Dirigent protein |
| NC_024466.2:151370 | LOC100192736 | -6.4419 | 4.83E-06 | 0.004071 | Zinc finger CCCH domain- |

| | | | | | |
|---|---|---|---|---|---|
| 661-151372724(-) | | 4 | | | containing protein 14 |
| NC_024467.2:20402828-20403920(-) | LOC100193182 | -8.59519 | 6.52E-08 | 0.000128 | Early nodulin-93 |
| NC_024459.2:186945224-186946587(+) | LOC103641577 | -4.49478 | 3.40E-07 | 0.000586 | Dirigent protein |
| NC_024468.2:96937763-96938529(+) | LOC103641458 | -4.5647 | 4.36E-07 | 0.000721 | Bowman-Birk type trypsin inhibitor |
| NC_024460.2:12565621-12567188(+) | aox3 | -4.92598 | 5.45E-07 | 0.000865 | Ubiquinol oxidase (EC 1.10.3.11) |
| NC_024465.2:56865886-56867259(-) | LOC103632406 | -7.31188 | 6.91E-07 | 0.001058 | Chlorophyllase1 |
| NC_024463.2:197531995-197533772(+) | LOC100281892 | -4.63142 | 9.96E-07 | 0.001469 | Beta-expansin 4 |

**Supplementary Table S6:** Selected top 20 up-regulated genes in DMI 56 leaf under low-N stress compared to its control (sufficient N) (named DMI 56_SN+ vs DMI 56_SN-).

| **DMI 56_SN+vs DMI_56SN-** | | | | | |
|---|---|---|---|---|---|
| **ID** | **Gene I'D** | **$Log_2$ fold change** | **P value** | **FDR** | **Gene Description** |
| NC_024466.2:113319211-113321231(-) | LOC103635712 | 8.807695 | 7.31E-10 | 5.19E-07 | carbohydrate metabolic process [GO:0005975] |
| NC_024461.2:138093686-138100193(-) | LOC103650615 | 8.125569 | 6.39E-19 | 8.63E-15 | protein suppressor of gene silencing 3 homolog |
| NC_024467.2:37432851-37435462(+) | LOC103638277 | 7.708985 | 6.41E-09 | 3.37E-06 | microtubule-based movement [GO:0007018] |
| NC_024459.2:174658843- | LOC103645065 | Inf | 1.48E- | 1.46E- | ubiquitin protein ligase |

| | | | | | |
|---|---|---|---|---|---|
| 174660244(+) | | | 10 | 07 | activity [GO:0061630] |
| NC_024463.2:203485460-203489985(-) | LOC103627571 | Inf | 1.85E-08 | 8.82E-06 | Plant elicitor peptide 4, Protein NRT1/ PTR FAMILY 7.2 (Protein NRT1/ PTR FAMILY 7.3) |
| NC_024461.2:146542784-146543596(-) | LOC103650692 | 8.011858 | 4.01E-16 | 1.81E-12 | AP2-EREBP transcription factor |
| NC_024461.2:138805064-138814004(+) | LOC103650620 | 6.974394 | 4.94E-16 | 2.00E-12 | D8Ertd354e protein |
| NC_024463.2:201022527-201023593(+) | LOC100284491 | 6.307505 | 9.09E-14 | 2.05E-10 | Dehydration-responsive element-binding protein 1A |
| NC_024467.2:60442035-60447747(+) | LOC103638554 | 6.096847 | 1.97E-13 | 4.20E-10 | Interferon-related developmental regulator family |
| NC_024462.2:150629045-150631230(+) | LOC100191789 | 6.606741 | 3.70E-13 | 6.82E-10 | oxidation-reduction process [GO:0055114] |
| NC_024465.2:101004710-101009817(-) | LOC103632640 | 6.563266 | 4.55E-12 | 6.82E-09 | Transducin family protein |
| NC_024463.2:216589025-216592463(-) | LOC100383210 | 5.773044 | 7.06E-12 | 9.34E-09 | oxidoreductase activity [GO:0016491] |
| NC_024461.2:232951331-232954396(-) | LOC103651649 | 8.427881 | 7.42E-12 | 9.40E-09 | Uncharacterized protein#integral component of membrane [GO:0016021] |
| NC_024468.2:71754071-71755095(-) | LOC103642522 | 5.628917 | 2.57E-11 | 3.06E-08 | Uncharacterized protein |
| NC_024466.2:104190500-104193277(+) | LOC100284502 | 5.769391 | 4.90E-11 | 5.52E-08 | Universal stress protein family protein |
| NC_024460.2:39565956-39567856(+) | LOC103646320 | 6.676106 | 1.04E-10 | 1.14E-07 | Zinc knuckle (CCHC-type) family protein |
| NC_024464.2:65322405- | DBP4 | 5.216594 | 1.29E- | 1.35E- | Dehydration-responsive element- |

| | | | | | |
|---|---|---|---|---|---|
| 65323578(+) | | | 10 | 07 | binding protein 1C |
| NC_024465.2:10857307-10858459(-) | LOC100280478 | 6.769611 | 6.38E-10 | 5.03E-07 | xylan catabolic process [GO:0045493] |
| NC_024463.2:7406160-7408459(-) | LOC100194167 | Inf | 2.04E-10 | 1.97E-07 | Transcriptional adapter ADA2b |
| NC_024461.2:172146140-172147330(+) | LOC100279664 | Inf | 2.22E-10 | 2.09E-07 | Uncharacterized protein |

**Supplementary Table S7:** Selected top 20 down-regulated genes in DMI 56 leaf under low-N stress compared to its control (sufficient N) (named DMI 56_SN+ vs DMI 56_SN-).

| **DMI 56_SN+ vs DMI 56_SN-** | | | | | |
|---|---|---|---|---|---|
| **ID** | **Gene I'D** | **$Log_2$ fold change** | **P value** | **FDR** | **Gene Description** |
| NC_024464.2:28923624-28924298(-) | LOC103630947 | -8.10065 | 4.35E-10 | 3.69E-07 | uncharacterized protein LOC103630947 |
| NC_024459.2:282687219-282688648(+) | LOC103644023 | -8.05002 | 3.91E-07 | 0.000117 | Regulator of Vps4 activity in the MVB pathway protein transport |
| NC_024466.2:171431000-171436167(+) | LOC100382585 | -9.30495 | 1.30E-15 | 4.80E-12 | Uncharacterized protein |
| NC_024463.2:72705848-72708598(-) | LOC109939704 | -7.70979 | 1.75E-15 | 5.90E-12 | HAT family dimerisation domain containing protein |
| NC_024468.2:142619140-142622387(-) | LOC100279863 | -7.74016 | 1.00E-08 | 5.00E-06 | Uncharacterized protein |
| NC_024466.2:162651852-162658240(+) | LOC103636279 | -6.91398 | 3.51E-15 | 1.02E-11 | Putative HLH DNA-binding domain superfamily protein |
| NC_024461.2:140251533-140253626(+) | LOC103650635 | -6.65397 | 5.18E-15 | 1.40E-11 | Amino acid permease 2 |

| | | | | | |
|---|---|---|---|---|---|
| NC_024463.2:65058208-65059377(+) | pco104428a | -7.01388 | 5.40E-14 | 1.37E-10 | Basic endochitinase 1 |
| NC_024465.2:3653592-3654276(+) | prp4 | -6.17621 | 6.37E-14 | 1.52E-10 | Pathogenesis related protein4 |
| NC_024461.2:18481283-18484038(+) | LOC103649793 | -7.78747 | 3.05E-13 | 6.18E-10 | Uncharacterized protein |
| NC_024466.2:167594748-167596284(+) | LOC103636366 | -8.80625 | 3.26E-13 | 6.28E-10 | DUF506 family protein |
| NC_024466.2:171234104-171236206(-) | LOC100383726 | -7.30156 | 7.15E-12 | 9.34E-09 | C0PIK5 |
| NC_024464.2:28928493-28931803(+) | LOC103629187 | -6.02827 | 7.54E-13 | 1.33E-09 | Nucleolin 2 |
| NC_024461.2:178451617-178453328(-) | LOC100272433 | -7.92669 | 8.56E-13 | 1.44E-09 | DBD_Tnp_Mut domain-containing protein |
| NC_024459.2:45286561-45290000(-) | asn3 | -6.79721 | 1.68E-12 | 2.72E-09 | Asparagine synthetase |
| NC_024468.2:122393248-122395555(-) | LOC109942912 | -7.31507 | 4.37E-10 | 3.69E-07 | Inter-alpha-trypsin inhibitor heavy chain-related |
| NC_024461.2:231817612-231818726(+) | LOC100191744 | -6.54629 | 5.75E-12 | 8.32E-09 | Uncharacterized protein |
| NC_024462.2:181681444-181682786(-) | LOC109945878 | -7.1094 | 8.59E-07 | 0.000238 | zinc finger BED domain-containing protein RICESLEEPER 3 |
| NC_024464.2:28920057-28921765(+) | LOC109940384 | -6.62009 | 5.35E-09 | 2.96E-06 | cytochrome P450 714C3 |
| NC_024460.2:219038973-219039945(+) | LOC109944405 | -7.085938687 | 3.68E-09 | 5.05E-03 | wiskott-Aldrich syndrome protein homolog 1-like |

**Supplementary Table S8:** Selected top 20 up-regulated genes in DMI 81 leaf under low-N stress compared to its control (sufficient N) (named DMI 81_SN+ vs DMI 81_SN-).

| **DMI 81_SN+ vs DMI 81_SN-** | | | | | |
|---|---|---|---|---|---|
| **ID** | **Gene I'D** | **$Log_2$ fold change** | **P value** | **FDR** | **Gene Description** |
| NC_024460.2:19078363-19082101(-) | LOC103646079 | 5.975173 | 9.98E-19 | 3.13E-15 | Prolyl oligopeptidase family protein |
| NC_024466.2:72403032-72404407(+) | MYBR87 | 7.733015 | 7.16E-18 | 1.94E-14 | MYB-related transcription factor |
| NC_024464.2:4037971-4039290(-) | LOC100275523 | 5.574258 | 1.69E-17 | 4.31E-14 | DUF4033 domain-containing protein |
| NC_024459.2:224454964-224456636(-) | LOC100382444 | 5.376252 | 1.63E-15 | 3.32E-12 | Glycosyltransferase |
| NC_024463.2:19357180-19359209(+) | LOC103626061 | 8.02514 | 3.58E-15 | 6.94E-12 | Cytochrome P450 CYP79A33 |
| NC_024460.2:174867030-174869050(-) | LOC100194073 | 5.653442 | 1.84E-14 | 3.25E-11 | UDP-glycosyltransferase 76C1 |
| NC_024465.2:181006315-181007733(-) | LOC109941001 | 4.741017 | 3.34E-14 | 5.44E-11 | Uncharacterized protein |
| NC_024461.2:215275386-215276968(+) | LOC103651394 | 4.712516 | 4.27E-14 | 6.68E-11 | MYB-related transcription factor |
| NC_024461.2:230035429-230038824(-) | LOC109944895 | 5.439925 | 1.81E-13 | 2.63E-10 | High affinity nitrate transporter 2.7 |
| NC_024466.2:153108122-153110782(-) | MYBR115 | 5.310249 | 5.43E-13 | 7.63E-10 | Protein RADIALIS-like 3 |
| NC_024465.2:149842408-149844850(-) | LOC100280965 | 4.929006 | 6.75E-13 | 9.16E-10 | CDGSH iron-sulfur domain |
| NC_024464.2:169245888-169247273(+) | gpm881 | 6.01495 | 8.35E-13 | 1.10E-09 | Protein RADIALIS-like 1 |
| NC_024461.2:29625385-29627271(-) | LOC100274601 | Inf | 1.87E-12 | 2.30E-09 | Exonuclease domain-containing protein |

| | | | | | |
|---|---|---|---|---|---|
| NC_024459.2:8372130-8373575(+) | TIDP2778 | 3.936372 | 8.19E-11 | 9.02E-08 | GATA zinc finger domain-containing protein 10 |
| NC_024460.2:50474639-50478074(-) | IDP1415 | 3.970469 | 1.04E-10 | 1.11E-07 | NA |
| NC_024468.2:823896-825905(-) | fer2 | 3.645845 | 7.12E-10 | 6.31E-07 | Ferritin (EC 1.16.3.1) |
| NC_024464.2:172322009-172324235(-) | LOC103630810 | 4.602535 | 1.16E-10 | 1.21E-07 | RNA pol sigma factor sigE chloroplastic/mitochondrial |
| NC_024468.2:3630797-3632942(+) | LOC103640724 | 3.827819 | 1.68E-10 | 1.71E-07 | Putative WRKY DNA-binding domain superfamily |
| NC_024468.2:19502940-19504931(-) | LOC103640919 | 4.577429 | 2.28E-10 | 2.21E-07 | Delta(12)-fatty-acid desaturase |
| NC_024461.2:232951331-232954396(-) | LOC103651649 | Inf | 2.43E-10 | 2.30E-07 | Uncharacterized protein |

**Supplementary Table S9:** Selected top 20 down-regulated genes in DMI 81 leaf under low-N stress compared to its control (sufficient N) (named DMI 81_SN+ vs DMI 81_SN-).

| **DMI 81_SN+ vs DMI81_SN-** | | | | | |
|---|---|---|---|---|---|
| **ID** | **Gene I'D** | **$Log_2$ fold change** | **P value** | **FDR** | **Gene Description** |
| NC_024463.2:195832851-195835981(-) | ys1 | -7.21435 | 4.35E-11 | 4.93E-08 | Iron-phytosiderophore transporter yellow stripe 1 (ZmYS1) |
| NC_024461.2:132281202-132282700(-) | phyS11 | -6.87036 | 1.94E-07 | 9.06E-05 | Phytase |
| NC_024461.2:47342404-47360743(-) | LOC103650045 | -10.0821 | 5.06E-28 | 6.86E-24 | protein ZINC INDUCED FACILITATOR-LIKE 1 |

| | | | | | |
|---|---|---|---|---|---|
| NC_024466.2:171234104-171236206(-) | LOC100383726 | -11.9543 | 2.65E-27 | 2.70E-23 | Uncharacterized protein |
| NC_024460.2:174319805-174320236(-) | LOC100384429 | -9.87882 | 1.08E-26 | 8.79E-23 | NA |
| NC_024468.2:25871996-25872962(-) | LOC103648196 | -7.22347 | 4.22E-23 | 2.86E-19 | Glutaredoxin-C13 |
| NC_024466.2:162651852-162658240(+) | LOC103636279 | -12.4476 | 5.90E-23 | 3.18E-19 | Putative HLH DNA-binding domain |
| NC_024459.2:81672408-81675503(-) | LOC100285480 | -5.93074 | 5.32E-07 | 0.000209 | Actin-depolymerizing factor |
| NC_024461.2:191129385-191130502(-) | LOC100279082 | -6.89655 | 1.12E-22 | 4.57E-19 | Uncharacterized protein |
| NC_024459.2:45286561-45290000(-) | asn3 | -6.82001 | 1.60E-20 | 5.91E-17 | Asparagine synthetase |
| NC_024461.2:149339751-149371343(+) | LOC103650720 | -6.37456 | 4.99E-20 | 1.69E-16 | Prpol, RNA-DNA hybrid ribonuclease activity [GO:0004523]; |
| NC_024459.2:275610625-275611161(+) | umc1290 | -6.34933 | 2.41E-18 | 7.02E-15 | Uncharacterized protein |
| NC_024463.2:65058208-65059377(+) | pco104428a | -5.76077 | 2.76E-17 | 6.62E-14 | Basic endochitinase 1 |
| NC_024464.2:166878688-166882923(+) | LOC103630691 | -5.63793 | 3.85E-17 | 8.71E-14 | Wall-associated kinase |
| NC_024461.2:58127334-58128164(-) | LOC109945000 | -5.19518 | 1.35E-15 | 2.89E-12 | Uncharacterized protein |
| NC_024466.2:167594748-167596284(+) | LOC103636366 | -5.68049 | 1.29E-14 | 2.39E-11 | NA |
| NC_024467.2:140318846-140322386(+) | asn4 | -6.69697 | 2.07E-12 | 2.47E-09 | DUF506 family protein |

| | | | | | |
|---|---|---|---|---|---|
| NC_024464.2:144377375-144377968(-) | LOC103630251 | -5.64386 | 0.000153 | 0.021183 | putative CRAL/TRIO domain containing, Sec14p-like phosphatidylinositol transfer family protein isoform X1 |
| NC_024465.2:181798111-181799403(+) | kin1 | -4.91294 | 1.01E-13 | 1.52E-10 | Extracellular ribonuclease LE |
| NC_024468.2:25661576-25662576(-) | LOC103640963 | -4.37758 | 8.98E-13 | 1.14E-09 | Glutaredoxin-C13 |

**Supplementary Table S10**: **Mapping percentage with reference B73 (version 4) genome**

| Genotype | Tissue | Stress treatment | Sample code | Mapping % |
|---|---|---|---|---|
| DMI 56 | Root | N- | 56_RN- | 93.25% |
| DMI 56 | Root | N+ | 56_RN+ | 88.10% |
| DMI 56 | leaf | N- | 56_SN- | 97.03% |
| DMI 56 | leaf | N+ | 56_SN+ | 96.83% |
| DMI 81 | Root | N- | 81_RN- | 94.63% |
| DMI 81 | Root | N+ | 81_RN+ | 95.91% |
| DMI 81 | leaf | N- | 81_SN- | 95.55% |
| DMI 81 | leaf | N+ | 81_SN+ | 97.55% |

Letter N+, and N- represent sufficient nitrogen, and nitrogen-deficiency conditions, respectively.

**Supplementary Table S11**: List of shortlisted genes with their primer sequences for validation through qRT-PCR.

| **S.No.** | **Gene Name** | **Primer name** | **Primer sequence** |
|---|---|---|---|
| 1 | Asparagine synthetase | XM_008659820.2_F | ACGTGTCTGTGTAGTGCCTC |
| | | XM_008659820.2_R | GCTCGGACAGAAAATGCCTG |

| | | | |
|---|---|---|---|
| 2 | High affinity transporter 2.3 | _XM_008658573.2_F | TACAAGACGGAGACGGGGAT |
| | | XM_008658573.2_R | GCGGTGTTGAAACCCTTGTC |
| 3 | Nodulin-related protein 1_ | NM_001165453.1_F | CAAGTCGTCGGAGGGGTTC |
| | | NM_001165453.1_R | CATCTTAAACAGCCCACCGC |
| 4 | Basic endochitinase | NM_001137022.1_F | ATACGGCCTGACCACGAAC |
| | | NM_001137022.1_R | TCCGTAAGGCTTCTGGCTC |
| 5 | Amino acid permease 3 | XM_008676200.2_F | CGTTCGCCCTCAGCCCTTT |
| | | XM_008676200.2_R | TGTACATCTCGACGGGGAAGT |
| 6 | Glutathione transferase 31 | NM_001279823.2_F | GTGGGCTGATCGTCTGTGTG |
| | | NM_001279823.2_R | AGGCTTGGAGAAGGAGCCAT |
| 7 | Transcription factor MYB 36 | XM_008671987.3_F | CTACGCAGGAGGTAGAGTGC |
| | | XM_008671987.3_R | CTACTCTCAAACGTCGCGGA |
| 8 | Transcription factor AP2-EREBP | XM_008676258.2_F | TCTTTGACATGCCAGGGCTC |
| | | XM_008676258.2_R | GTGCAGTCGACGTGGCTAT |
| 9 | Nitrate transport1: | NM_001279422.1_F | ATCTTCGGGGTCATCCCCTT |
| | | NM_001279422.1_R | GTGCACGAACACCACAGGTA |
